# Impact of anxiety and depression across childhood and adolescence on adverse outcomes in young adulthood: a UK birth cohort study

**DOI:** 10.1192/bjp.2023.23

**Published:** 2023-05

**Authors:** Isabel Morales-Muñoz, Pavan K. Mallikarjun, Joht S. Chandan, Rasiah Thayakaran, Rachel Upthegrove, Steven Marwaha

**Affiliations:** Institute for Mental Health, School of Psychology, University of Birmingham, UK; Early Intervention Service, Birmingham Women's and Children's NHS Trust, Birmingham, UK; Institute of Applied Health Research, University of Birmingham, UK; Institute for Mental Health, School of Psychology, University of Birmingham, UK and Early Intervention Service, Birmingham Women's and Children's NHS Trust, Birmingham, UK; Institute for Mental Health, School of Psychology, University of Birmingham, UK; Early Intervention Service, Birmingham Women's and Children's NHS Trust, Birmingham, UK; Specialist Mood Disorders Clinic, Zinnia Centre, Birmingham, UK; and The Barberry National Centre for Mental Health, Birmingham, UK

**Keywords:** Anxiety, depression, comorbidity, adverse outcome, longitudinal cohort

## Abstract

**Background:**

Little is still known about the long-term impact of childhood and adolescent persistent depression and anxiety in adulthood.

**Aims:**

To investigate the impact of persistent anxiety, depression, and comorbid anxiety and depression across childhood and adolescence on the development of multiple adverse outcomes in young adulthood.

**Method:**

This study used data from 8122 participants in the Avon Longitudinal Study of Parents and Children cohort. The Development and Well-Being Assessment (DAWBA) examined child anxiety and depression symptomatology. The DAWBA generalised anxiety and mood subscales at 8, 10 and 13 years were selected, and a measure of comorbid anxiety and depression symptoms was created at each time point. Further, several mental and physical health, substance misuse and education/employment problems were assessed at 24 years. Latent class growth analyses were used to detect trajectories of anxiety, depression and comorbid anxiety and depression; and logistic regression to examine how persistent anxiety, depression or both were associated with adverse outcomes at 24 years.

**Results:**

All three classes with persistent anxiety, depression or both were significantly associated with presenting with any mental health problems and any education/employment problem. Persistent high levels of depression and high levels of comorbid anxiety and depression, but not persistent high anxiety, were significantly associated with any physical health problem. High levels of comorbid anxiety and depression was the only DAWBA domain significantly associated with substance misuse; and overall, this was the domain that exerted the greatest negative impact, as it presented the highest odd ratio values.

**Conclusions:**

Children and adolescents with comorbid anxiety and depression are at the highest risk for having more adverse outcomes at 24 years.

## Background

Depression and anxiety are the largest contributors to the health burden experienced by children and adolescents.^1^ According to a meta-analysis, 12.3% meet criteria for anxiety disorder in middle childhood and 11.0% in adolescence,^2^ and major depression has an estimated prevalence of 4% in school-aged children^3^ and 7.5% in adolescents.^4^ Overall, anxiety and depression disorders affect a significant number of children and adolescents worldwide.^5^ Further, a recent analysis by the Global Burdens of Disease study group found that among young people aged 10–24 years in Europe, anxiety and depressive disorders contributed to a substantial amount of years lived with disability, reporting 655 912 and 626 008 years, respectively.^6^

Importantly, anxiety and depression are highly comorbid with each other, especially in children and adolescents.^7^ About 25–50% of youth with depression have comorbid anxiety and about 10–15% of youth with anxiety have depression.^8^ The presence of comorbid anxiety and depression matters because this has an impact on their treatment and outcomes. For instance, patients with anxious major depressive disorder, compared with patients with non-anxious major depressive disorder, have more severe impairments and more suicidal ideation.^9^ Similarly, comorbid anxiety and depression predicts worse outcomes.^10^

Longitudinal studies suggest that when left untreated, anxiety and depression can put children and adolescents at risk for long-term adverse outcomes, including substance misuse, unemployment and/or mental health difficulties.^11,12^ Early identification and effective treatment of anxiety and depression in children and adolescents are therefore crucial. To date, there is limited longitudinal research investigating how childhood and adolescent anxiety and/or depression may have an impact on long-term outcomes in adulthood. Among the existing evidence, a longitudinal cohort study reported that childhood anxiety disorder was associated with adverse functioning in health, financial and/or interpersonal outcomes in young adulthood.^11^ The same longitudinal cohort recently reported that childhood and adolescent depression was associated with broad effects on psychiatric problems, substance misuse and functional outcomes in adulthood.^13^ However, these authors only focused on the longitudinal patterns of anxiety and depression separately, and comorbid anxiety and depression was not explored. Further, their sample size was relatively small (i.e. *n* = 1420), compared with other currently available longitudinal cohort studies (e.g. Avon Longitudinal Study of Parents and Children (ALSPAC)).

To date, a small number of studies characterising the trajectories of depression and anxiety across childhood and adolescence provide some evidence that there is a group of young people with elevated and persistent anxiety and/or depression levels.^14–18^ A recent longitudinal cohort study reported that different trajectories of depression throughout childhood and adolescence were associated with worse adult educational and employment outcomes,^19^ but other areas in adulthood were not explored.

## Aims

The current study, therefore, aimed to fill this knowledge gap. The overall aim of this study was to investigate the impact of persistent anxiety and depression across childhood and adolescence on the development of multiple outcomes in young adulthood, including mental and physical health, substance misuse and education/employment problems.

Our objectives were to:

- examine how anxiety, depression and comorbid anxiety and depression develop over time from 8 to 13 years old;
- investigate whether and how persistent high levels of anxiety, depression and comorbid anxiety and depression associate with multiple outcomes at 24 years; and
- examine which of these domains (i.e. anxiety, depression or comorbid anxiety and depression) across childhood and adolescence exert a greater impact on adverse outcomes at 24 years.

We hypothesised that those children presenting comorbid anxiety and depression over time would show more severe adverse outcomes in young adulthood, compared with those children presenting with anxiety or depression alone.

## Method

### Participants

The ALSPAC is a UK birth cohort study examining the determinants of development, health and disease during childhood and beyond.^20^ Pregnant women resident in Avon with expected dates of delivery 1 April 1991 to 31 December 1992 were invited to participate. The ALSPAC study website contains details of all the data available (http://www.bristol.ac.uk/alspac/researchers/our-data/). The initial number of pregnancies enrolled was 14 541. Further details of this cohort appear in Supplementary Appendix 1 (available at https://doi.org/10.1192/bjp.2023.23).

The authors assert that all procedures contributing to this work comply with the ethical standards of the relevant national and institutional committees on human experimentation and with the Helsinki Declaration of 1975, as revised in 2008. All procedures involving human patients were approved by the ALSPAC Law and Ethics Committee and local research ethics committees. Full details of the ALSPAC consent procedures and the corresponding approval numbers at each time point are available on the study website (http://www.bristol.ac.uk/alspac/researchers/research-ethics/). Written informed consent was obtained from participants (parents and/or the children, when applicable). We used the Strengthening the Reporting of Observational Studies in Epidemiology cohort reporting guidelines.

### Anxiety and depression measures

The Development and Well-Being Assessment (DAWBA)^21^ was administered as a parent-report questionnaire to capture child and adolescent psychopathology. Further details of the DAWBA appear in Supplementary Appendix 2.

To calculate anxiety and depression trajectories across childhood, we focused on the DAWBA generalised anxiety and mood subscales at 8, 10 and 13 years. We selected these time points because (a) these were the assessment points available in ALSPAC for parent-reported DAWBA: and (b) they cover the early adolescence period, which is a critical period for brain maturation and thus the emergence of subsequent psychopathology.

Regarding anxiety, we created a composite measure of generalised anxiety, by calculating the arithmetic mean of: (a) generalised anxieties total score that was calculated using the generalised anxieties subscale; and (b) generalised anxieties symptoms score that was calculated using the generalised anxieties symptoms subscale. To capture depression symptoms trajectories, we used the mood total score, from the mood subscale. Finally, we created a measure of comorbid anxiety and depression (i.e. composite measure of generalised anxiety + the mood total score). This was done at each time point, separately.

A description of each of the items comprising the DAWBA is detailed in Supplementary Table 1).

### Outcomes at 24 years

Mental health problems at 22–24 years included presenting psychotic disorder, severe depression, generalised anxiety disorder, social phobia, specific phobia and panic disorder at 24 years, and/or lifetime hypomania at 22–23 years. Psychotic disorder was measured using the semi-structured Psychosis-Like Symptom Interview;^22^ hypomania using the Hypomania Checklist;^23^ and severe depression, generalised anxiety disorder, social phobia, specific phobia and panic disorder using a self-administered online version of the Clinical Interview Schedule-Revised.^24^ Further details of these specific measures are included in Supplementary Table 2).

Physical health problems at 24 years included having any of the following medical conditions for at least 6 months, at 24 years old: diabetes, asthma, arthritis, heart problems, stroke or cancer and/or kidney disease. These conditions were directly asked of participants as follows using a single item by ALSPAC researchers during data collection: do you have any of the following conditions (diabetes, asthma, arthritis, heart problems, stroke or cancer and/or kidney disease)? Further, obesity at 24 years was included using body mass index, which was categorised as obesity (≥30.0 kg/m^2^) versus non-obesity (<30.0 kg/m^2^), following the World Health Organization guidelines.^25^ Finally, sleep problems at 24 years were assessed by self-reported questionnaire, with the following item: ‘In past month, I had problems getting to sleep or back to sleep’.

Substance misuse at 24 years included presenting alcohol and/or cannabis misuse at 24 years. We decided to focus only on alcohol and cannabis misuse, and not on other additional illicit drugs, as alcohol and cannabis are the most commonly used substances among young adults. Alcohol misuse at 24 years was measured using the DSM-IV tool^26^ (further description appears in Supplementary Table 3). Cannabis use at 24 years was measured using the Cannabis Abuse Screening Test (CAST) questionnaire,^27^ and a cut-off score of 4 was selected to detect those individuals with cannabis misuse.

Education/employment status and problems at 24 years were coded as: not in education, employed or in training scheme; difficulty keeping up with coursework/studies; or difficulty keeping up with work; again, these were asked about by a direct question to participants.

### Confounders

Multiple early-life family risk factors were assessed using the Family Adversity Index (FAI) during pregnancy, and at the child's 2 and 4 years of life. The FAI comprises 18 mother-reported items on early parenthood, housing conditions, maternal education, financial difficulties, parents’ relationship, family size, family major problems, maternal psychopathology, parents’ substance misuse, crime records, partner support and social network. Points were summed at each time point for a total FAI score across the three time points. Total FAI score was included as a confound as early-life adversities are associated with an increased risk of depression and anxiety.

Other covariates, which are known to have an impact in youth mental health, were child's gender (male versus female), gestational age (weeks) and ethnicity (White versus Black and minority ethnic), and maternal age when baby was born (years). For the health outcomes at 24 years, we also controlled for parent-reported child's health (i.e. good versus bad) at 4 weeks, and at 8, 10 and 13 years, as poor physical health in childhood associates with a variety of negative health-related outcomes in adulthood.

### Statistical analysis

First, latent class growth analyses (LCGA) were conducted using Mplus v8, to detect trajectories of anxiety, depression and comorbid anxiety and depression, separately, across childhood and adolescence. Several models were fitted by increasing the number of classes. The best fitting classification model was chosen according to fit indices (i.e. Bayesian information criteria (BIC) and Vuong–Lo–Mendell–Rubin (VLMR) test). Lower BIC values suggest better model fit. A significant VLMR value suggests that a *K*-class model fits the data better than a (*K* − 1) class model. Entropy was also used to select the best model fit; entropy with values approaching 1 indicates clear delineation of classes. Finally, to decide the optimal class solution, an emphasis was placed on large enough group sizes (i.e. >2% of the sample) and clinically relevant and informative interpretation, according to the previous literature on the topic. Missing values because of attrition were handled by the full-information maximum-likelihood estimation method.

The second stage analysis was conducted in SPSS-v27 to investigate the prospective associations between persistent high levels of anxiety, persistent high levels of depression, and persistent high levels of comorbid anxiety and depression, identified by LCGA and adverse outcomes at 24 years. The derived latent classes from the LCGA were included as predictor (with the class with the highest number of ‘cases’, as the reference) and adverse outcomes at 24 years as outcome. We conducted logistic regression analysis for each exposure and each outcome, in separate analysis. For each of the analyses, we controlled for FAI, gender, ethnicity, gestational age and maternal age at birth. For the health-related outcomes at 24 years, we also included parent-reported child's health at 4 weeks, and at 8, 10 and 13 years as covariates.

To deal with missingness, which was unlikely to be occurring at random (i.e. our missing data was systematically related to the unobserved data), we conducted logistic regression to identify significant factors associated with attrition, using inverse probability weights. Characteristics associated with attrition at 24 years old were being a boy, younger mother, shorter gestational age, less weight at birth and higher socioeconomic levels (Supplementary Table 4). Using the variables associated with selective dropout as the factors, we fitted a logistic regression model to determine weights for each individual using the inverse probability of response. The regression coefficients from this model were used to determine probability weights for the covariates in the primary analyses.

## Results

Data were available on 8122 participants who reported on the DAWBA at 8, 10 and 13 years. A description of the sociodemographic, DAWBA and outcome variables at 24 years old are presented in Tables 1 and 2.

**Table 1:** Description of the sociodemographic, the Development and Well-Being Assessment variables and outcome at 24 years old

Variable	8 years old		10 years old		13 years old		24 years old		Health at different ages^a^	
									Good (%)	Poor (%)
Gender, boys/girls: *n*/*n* (%/%)	4235/4007	(51.4/48.6)	3938/3869	(50.4/49.6)	3462/3444	(50.1/49.9)	1458/2429	(37.5/62.5)	–	
Ethnicity, White/Black and minority ethnic: *n*/*n* (%/%)	7808/145	(98.2/1.8)	7106/125	(98.3/1.7)	6323/104	(98.4/1.6)	3405/78	(97.8/2.2)		
Birth weight, kg: mean (s.d.)	3.42	(0.55)	3.42	(0.54)	3.43	(0.54)	3.41	(0.53)		
FAI, total score: mean (s.d.)	4.06	(4.10)	3.93	(4.01)	3.86	(4.01)	3.61	(3.84)		
Maternal age at birth: mean (s.d.)	28.43	(4.82)	29.03	(4.57)	28.04	(4.68)	29.45	(4.56)		
Gestational age, weeks: mean (s.d.)	39.07	(2.39)	39.41	(1.85)	39.44	(2.31)	39.49	(1.80)		
Baby's health at 4 weeks, *n* (%)	–	–	–	–	–	–	–	–	3452 (99.6)	13 (0.4)
Child's health at 8 years, *n* (%)	–	–	–	–	–	–	–	–	3016 (98.9)	35 (1.1)
Child's health at 10 years, *n* (%)	–	–	–	–	–	–	–	–	2926 (98.3)	52 (1.7)
Child's health at 13 years, *n* (%)	–	–	–	–	–	–	–	–	2873 (98.7)	38 (1.3)
DAWBA generalised anxiety composite, mean (s.d.)	1.16	(1.59)	2.37	(1.61)	2.40	(1.71)	–	–		
DAWBA mood total score, mean (s.d.)	0.60	(1.43)	0.53	(1.42)	0.61	(1.61)	–	–		
DAWBA anxiety + depression, mean (s.d.)	1.61	(2.37)	1.06	(2.85)	1.22	(3.23)	–	–		

DAWBA, The Development and Well-Being Assessment; FAI, Family Adversity Index.

a. Child's health was reported by the parents at the ages of 4 weeks and 8, 10 and 13 years old.

**Table 2:** Description of the outcomes at 24 years old

Adverse outcomes at 24 years old	Yes (%)	No (%)
Any mental health problem	454 (11.7)	3413 (88.3)
Psychotic disorder	47 (1.2)	3842 (98.8)
Severe depression	53 (1.3)	3913 (98.7)
Generalised anxiety disorder	386 (9.8)	3571 (90.2)
Specific phobia	8 (0.2)	3950 (99.8)
Social phobia	5 (0.1)	3953 (99.9)
Panic disorder	36 (0.9)	3921 (99.1)
Any physical health problem	516 (13.3)	3351 (86.7)
Diabetes	20 (0.5)	3847 (99.5)
Asthma	434 (11.2)	3433 (88.8)
Arthritis	23 (0.6)	3844 (99.4)
Stroke/cancer	6 (0.2)	3861 (99.8)
Kidney disease	6 (0.2)	3861 (99.8)
Heart problems	128 (3.4)	3682 (96.6)
Obesity	508 (13.1)	3381 (86.9)
Sleep problems	1469 (37.2)	2475 (62.8)
Any substance misuse	444 (11.5)	3418 (88.5)
Alcohol misuse	344 (9.5)	3492 (90.5)
Cannabis misuse	100 (0.6)	3862 (99.4)
Any education/employment problem	458 (11.8)	3426 (88.2)
Not in education/employed/training	316 (8.1)	3568 (91.9)
Difficulties in keeping up with coursework	153 (9.9)	3481 (90.1)
Difficulties in keeping up with work	94 (2.9)	3104 (97.1)

### Latent classes of anxiety, depression, and comorbid anxiety and depression

Table 3 shows VLMR, BIC and entropy for all models assessed (two to six classes), tested for each of the three main DAWBA domains. BIC decreased with the addition of each class indicating a better model fit for more classes. This pattern is typically found in large samples.^28^ However, decreases in BIC became considerably smaller in the three-classes model compared with the two-classes model, which suggested a better model fit for the two-classes model (see Supplementary Fig 1).

**Table 3:** Bayesian Information Criterion (BIC), Vuong–Lo–Mendell–Rubin Likelihood Test (VLMR) *P-*values and entropy for classes two to six, for Development and Well-Being Assessment (DAWBA) anxiety + depression total score, from 8 to 13 years old

DAWBA composite scores of general anxieties	BIC	VLMR, *P*	Entropy
2 classes	47 499.75	<0.001	0.85
3 classes^a^	46 831.34	0.0014	0.86
4 classes	44 240.28	0.0016	0.82
5 classes	43 590.28	0.0481	0.85
6 classes	43 317.49	0.2610	0.83
DAWBA mood total score			
classes	69 180.71	<0.001	0.92
3 classes^a^	65 450.06	0.0294	0.94
4 classes	63 537.54	0.0820	0.91
5 classes	60 722.04	0.0372	0.91
6 classes	58 608.20	0.4439	0.90
Combined score of DAWBA anxiety + depression^b^			
2 classes	103 853.42	<0.001	0.84
3 classes^a^	100 079.46	<0.001	0.85
4 classes	97 359.17	0.0211	0.83
5 classes	97 362.40	0.9459	0.66
6 classes	94 166.58	0.7382	0.78

a. The three-classes model presented overall the best model fit values for each of the DABWA subscales tested.

b. Combined score of DAWBA anxiety + depression is DAWBA composite score of general anxieties + DAWBA mood total score (i.e. internalising symptoms).

Further, VLMR showed a statistically significant difference for the two-classes, three-classes, four-classes and five-classes models for DAWBA generalised anxiety and DAWBA depression, and VLMR was statistically significant for two-classes, three-classes and four-classes models for DAWBA anxiety + depression. The six-classes model did not offer a significant difference, for any of the three domains. Importantly, two-classes and three-classes models showed higher significant values for VLMR than the four-classes and five-classes models, suggesting a better model fit for two- or three-classes models, for each of the DAWBA domains.

**Fig. 1:**
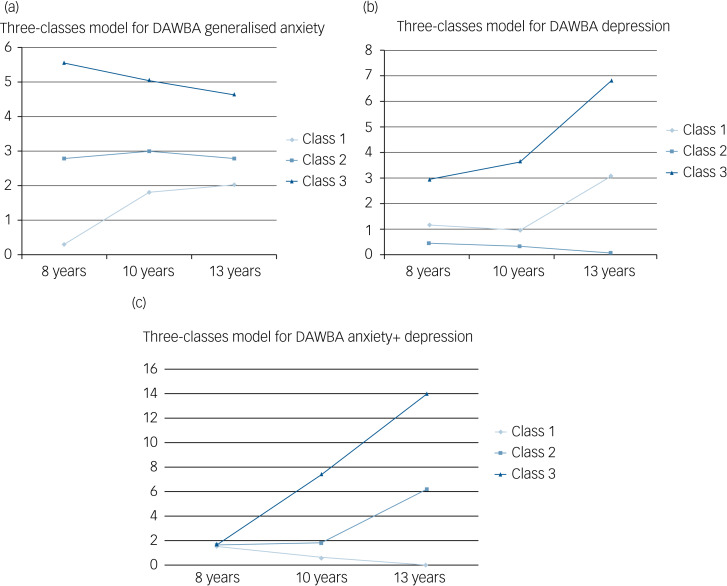
Growth trajectories of anxiety, depression, and comorbid anxiety and depression symptoms across childhood and adolescence. The latent class growth analyses detected a best model fit for three classes for the three domains. *x*-axis represents the three time points in childhood and adolescence, and the *y*-axis represents the mean total score of Development and Well-Being Assessment (DAWBA). (a) Reflects the trajectories for anxiety. Class 3 (dark-blue line) is characterised by persistent (and decreasing) high levels. Class 1 (light-blue line) represents persistent (and increasing) low levels. Finally, class 2 (mid-blue line) reflects persistent intermediate levels. (b) Shows the trajectories for depression. These trajectories show a class 3 (dark-blue line) characterised by persistent (and increasing) high levels; a class 1 (light-blue line) reflecting persistent (and increasing) intermediate levels; and finally, a class 2 (mid-blue line) representing persistent (and decreasing) low levels. Finally, (c) shows the trajectories for comorbid anxiety and depression. These trajectories show a class 3 (dark-blue line) characterised by persistent (and increasing) high levels; a class 1 (light-blue line) reflecting persistent (and decreasing) low levels; and finally, a class 2 (mid-blue line) representing persistent (and decreasing) intermediate levels. Further, the three classes of this specific domain seem to equally present low levels of symptomatology at 8 years, while the discrepancies between trajectories clearly appear by the age of 10 years.

Finally, the entropy values for the three-classes model for each of the DAWBA domains were the highest, compared with the other *n*-classes model (and all >0.80), indicating the highest classification accuracy for the three-classes model. Therefore, and overall, the three main fit indices tested (i.e. VLMR, BIC and entropy) suggested that for each of the DAWBA domains, the three-classes model offered the best model fit, followed by the two-classes model. To confirm this optimal three-classes solution, we checked that there were large enough group sizes (i.e. >2% of the sample) in each of the classes, which was the case; and that the trajectories detected were clinically relevant. In fact, the three-classes model was more clinically relevant than the two-classes model, as the two-classes model was not able to detect a group with moderate levels of symptomatology. This was corroborated by the clinical experts in our study (i.e. S.M. and R.U.). Therefore, a three-classes model offered overall the best fit, for each domain. The three derived classes of anxiety from the three-classes model, for each of the three DAWBA domains appear in Fig. 1.

The three-classes model of DAWBA generalised anxiety included: class 1 (*n* = 6331, 72.9%) characterised by persistent and increasing low levels, class 2 (*n* = 1882, 21.7%) characterised by persistent and decreasing intermediate levels, and class 3 (*n* = 469, 5.4%) characterised by persistent and decreasing high levels.

The three-classes model of DAWBA depression included: class 2 (*n* = 8324, 88.2%) characterised by persistent and decreasing low levels, class 1 (*n* = 695, 7.4%) characterised by persistent and increasing intermediate levels, and class 3 (*n* = 421, 4.5%) characterised by persistent and increasing high levels.

The three-classes model of combined DAWBA generalised anxiety and depression total scores included: class 1 (*n* = 11 154, 91.2%) characterised by decreasing low levels, class 2 (*n* = 703, 5.8%) characterised by increasing intermediate levels, and class 3 (*n* = 369, 3.0%) characterised by increasing high levels.

### Associations between persistent anxiety, persistent depression, and persistent comorbid anxiety and depression, with adverse outcomes at 24 years

For the logistic regressions between LCGA three-classes model and the adverse outcomes at 24 years, we focused on class 3, as it represented the individuals at highest risk, in each of the three domains. Our results showed that all three classes with persistent anxiety, depression or both were significantly associated with presenting any mental health problems at 24 years compared with the reference class (anxiety: odds ratio (OR) = 2.09, 95% CI 1.63–2.69, *P* < 0.001; depression: OR = 2.07, 95% CI 1.50–2.87, *P* < 0.001; comorbid: OR: 1.99, 95% CI 1.49–2.65, *P* < 0.001] (see Table 4). Further, persistent high levels of depression (OR = 1.27, 95% CI 1.09–1.48, *P* = 0.002) and high levels of comorbid anxiety and depression (OR = 1.40, 95% CI 1.21–1.62, *P* < 0.001), but not persistent high anxiety, were significantly associated with any physical health problem at 24 years (see Table 4).

**Table 4:** Associations between latent class growth analyses three-classes model and mental and physical health adverse outcomes at 24 years

Outcomne	OR	95% CI	*P*
*Any mental health problem at 24 years*			
DAWBA anxiety			
Anxiety Class1 (ref)	–	–	–
Anxiety Class2	1.07	0.91 to 1.26	0.417
Anxiety Class3	**2.09**	**1.63 to 2.69**	**<0**.**001**
Gender	**0**.**48**	**0.42 to 0.56**	**<0**.**001**
Gestational age	0.97	0.93 to 1.09	0.083
FAI total score	**1**.**04**	**1.03 to 1.06**	**<0**.**001**
Ethnicity	1.08	0.67 to 1.73	0.758
Maternal age birth	0.99	0.98 to 1.01	0.994
DAWBA depression			
Mood Class2 (ref)	–	–	–
Mood Class1	0.93	0.75 to 1.15	0.490
Mood Class3	**2**.**07**	**1.50 to 2.87**	**<0**.**001**
Gender	**0**.**52**	**0.46 to 0.60**	**<0**.**001**
Gestational age	0.97	0.94 to 1.01	0.111
FAI total score	**1**.**04**	**1.04 to 1.06**	**<0**.**001**
Ethnicity	1.01	0.63 to 1.62	0.969
Maternal age birth	0.99	0.98 to 1.01	0.336
DAWBA Anxiety + Depression			
A + D Class2 (ref)	–	–	–
A + D Class1	1.17	0.95 to 1.44	0.148
A + D Class3	**1**.**99**	**1.49 to 2.65**	**<0**.**001**
Gender	**0**.**52**	**0.45 to 0.59**	**<0**.**001**
Gestational age	0.98	0.95 to 1.02	0.289
FAI total score	**1**.**04**	**1.03 to 1.05**	**<0**.**001**
Ethnicity	1.01	0.63 to 1.62	0.959
Maternal age birth	0.99	0.98 to 1.01	0.376
*Any physical health problem at 24 years*			
DAWBA anxiety			
Anxiety Class1 (ref)	–	–	–
Anxiety Class2	1.11	0.99 to 1.23	0.059
Anxiety Class3	1.12	0.93 to 1.35	0.217
Gender	**0**.**46**	**0.42 to 0.50**	**<0**.**001**
Gestational age	1.00	0.98 to 1.03	0.827
FAI total score	**0**.**98**	**0.97 to 0.99**	**<0**.**001**
Ethnicity	1.07	0.77 to 1.48	0.693
Maternal age birth	**1**.**01**	**1.00 to 1.02**	**0**.**006**
Child´s health 4 weeks	0.00	0.00 to 0.00	0.998
Child´s health 8 years	**1**.**83**	**1.12 to 3.01**	**0**.**016**
Child´s health 10 years	**2**.**88**	**1.91 to 4.37**	**<0**.**001**
Child´s health 13 years	**1**.**92**	**1.13 to 3.25**	**0**.**015**
DAWBA depression			
Mood Class2 (ref)	–	–	–
Mood Class1	**1**.**23**	**1.02 to 1.48**	**0**.**033**
Mood Class3	**1**.**27**	**1.09 to 1.48**	**0**.**002**
Gender	**0**.**47**	**0.43 to 0.52**	**<0**.**001**
Gestational age	1.00	0.98 to 1.03	0.743
FAI total score	**0**.**98**	**0.97 to 0.99**	**<0**.**001**
Ethnicity	0.99	0.72 to 1.37	0.973
Maternal age birth	**1**.**01**	**1.00 to 1.02**	**0**.**010**
Child´s health 4 weeks	0.00	0.00 to 0.00	0.998
Child´s health 8 years	**1**.**92**	**1.17 to 3.14**	**0**.**009**
Child´s health 10 years	**2**.**81**	**1.86 to 4.27**	**<0**.**001**
Child´s health 13 years	**1**.**93**	**1.14 to 3.28**	**0**.**015**
DAWBA anxiety + depression			
A + D Class2 (ref)	–	–	–
A + D Class1	**1**.**28**	**1.04 to 1.56**	**0**.**017**
A + D Class3	**1**.**40**	**1.21 to 1.62**	**<0**.**001**
Gender	**0**.**48**	**0.44 to 0.52**	**<0**.**001**
Gestational age	1.01	0.98 to 1.03	0.580
FAI total score	0.98	0.97 to 0.99	<0.001
Ethnicity	0.95	0.69 to 1.30	0.732
Maternal age birth	**1**.**02**	**1.01 to 1.02**	**<0**.**001**
Child´s health4 weeks	0.00	0.00 to 0.00	0.998
Child´s health 8 years	**1**.**92**	**1.17 to 3.14**	**0**.**010**
Child´s health 10 years	**2**.**81**	**1.85 to 4.26**	**<0**.**001**
Child´s health 13 years	**1**.**91**	**1.12 to 3.22**	**0**.**017**

Significance level at <0.050 (bold). A + D = DAWBA Generalised anxiety composite score + DAWBA mood total score (i.e. internalising symptoms). FAI, Family adversity index.

In relation to substance misuse at 24 years, high levels of comorbid anxiety and depression was the only DAWBA domain significantly associated with this outcome (OR = 1.57, 95% CI 1.15–2.15, *P* < 0.001; see Table 5). Finally, all three classes with persistent anxiety, depression or both were significantly associated with presenting any education/employment problem at 24 years (i.e. anxiety: OR = 1.56, 95% CI 1.20–2.03, *P* = 0.001; depression: OR = 1.38, 95% CI 1.00–1.91, *P* = 0.047; comorbid: OR = 1.48, 95% CI 1.07–2.05, *P* = 0.018; see Table 5). Unadjusted logistic regressions (i.e. without covariates) were also reported for each of the main exposure and main outcome measures, which showed similar results to the adjusted models (see Supplementary Table 5).

**Table 5:** Associations between latent class growth analysis three-classes model and substance misuse and employment/educational adverse outcomes at 24 years

Outcome	OR	95% CI	*P*
*Substance misuse at 24 years*			
DAWBA anxiety			
Anxiety Class1 (ref)	–	–	–
Anxiety Class2	1.04	0.90 to 1.20	0.628
Anxiety Class3	0.83	0.62 to 1.11	0.210
Gender	**2**.**51**	**2.20 to 2.86**	**<0**.**001**
Gestational age	1.02	0.99 to 1.06	0.161
FAI total score	**1**.**03**	**1.02 to 1.05**	**<0**.**001**
Ethnicity	0.99	0.64 to 1.55	0.977
Maternal age birth	0.99	0.98 to 1.01	0.194
DAWBA depression			
Mood Class2 (ref)	–	–	–
Mood Class1	**1.44**	**1.18 to 1.86**	**0**.**031**
Mood Class3	1.32	0.99 to 1.92	0.071
Gender	**2**.**41**	**2.12 to 2.75**	**<0**.**001**
Gestational age	1.03	0.99 to 1.07	0.084
FAI total score	**1**.**02**	**1.01 to 1.03**	**0**.**003**
Ethnicity	1.01	0.65 to 1.56	0.908
Maternal age birth	0.99	0.98 to 1.01	0.480
DAWBA anxiety + depression			
A + D Class2 (ref)	–	–	–
A + D Class1	**1**.**51**	**1.25 to 1.82**	**0**.**005**
A + D Class3	**1**.**57**	**1.15 to 2.15**	**<0**.**001**
Gender	**2**.**36**	**2.08 to 2.68**	**<0**.**001**
Gestational age	1.03	0.99 to 1.07	0.057
FAI total score	**1**.**02**	**1.01 to 1.03**	**<0**.**001**
Ethnicity	1.00	0.64 to 1.55	1.00
Maternal age birth	1.00	0.99 to 1.02	0.719
*Any education/employment problem at 24 years*			
DAWBA anxiety			
Anxiety Class1 (ref)	–	–	–
Anxiety Class2	**1**.**27**	**1.10 to 1.47**	**0**.**001**
Anxiety Class3	**1**.**56**	**1.20 to 2.03**	**0**.**001**
Gender	0.89	0.78 to 1.01	0.083
Gestational age	**0**.**94**	**0.91 to 0.98**	**0**.**001**
FAI total score	**1**.**03**	**1.02 to 1.04**	**<0**.**001**
Ethnicity	0.92	0.57 to 1.50	0.747
Maternal age birth	1.10	0.99 to 1.12	0.207
DAWBA depression			
Mood Class2 (ref)	–	–	–
Mood Class1	**1**.**77**	**1.47 to 2.12**	**<0**.**001**
Mood Class3	**1**.**38**	**1.00 to 1.91**	**0**.**047**
Gender	**0**.**86**	**0.76 to 0.98**	**0**.**026**
Gestational age	**0**.**94**	**0.91 to 0.98**	**0**.**001**
FAI total score	**1**.**03**	**1.01 to 1.04**	**<0**.**001**
Ethnicity	1.04	0.66 to 1.65	0.854
Maternal age birth	1.01	0.99 to 1.03	0.114
DAWBA anxiety + depression			
A + D Class2 (ref)	–	–	–
A + D Class1	**1**.**68**	**1.40 to 2.01**	**<0**.**001**
A + D Class3	**1**.**48**	**1.07 to 2.05**	**0**.**018**
Gender	**0**.**86**	**0.76 to 0.98**	**0**.**022**
Gestational age	**0**.**96**	**0.92 to 0.99**	**0**.**009**
FAI total score	**1**.**03**	**1.02 to 1.04**	**<0**.**001**
Ethnicity	1.02	0.64 to 1.62	0.926
Maternal age birth	1.01	0.99 to 1.02	0.198

Significance level at <0.050 (bold). A + D, DAWBA Generalised anxiety composite score + DAWBA mood total score (i.e. internalising symptoms); FAI, Family adversity index.

We additionally examined the logistic regression with each individual outcome at 24 years. A detailed description of these results can be found in Supplementary Table 6 for individual mental health outcomes; Supplementary Table 7 for individual physical health outcomes; Supplementary Table 8 for sleep problems; Supplementary Table 9 for individual functional outcomes; and Supplementary Table 10 for individual substance misuse outcomes. Briefly, all three classes with persistent anxiety, depression or both were significantly associated with psychotic disorder (highest OR for comorbid anxiety and depression), severe depression (highest OR for comorbid anxiety and depression), generalised anxiety disorder (highest OR for depression) and panic disorder (highest OR for anxiety), all at 24 years.

Concerning individual physical health outcomes at 24 years, persistent high anxiety was associated with diabetes, persistent high depression and high comorbid anxiety and depression symptoms with asthma and arthritis (highest OR for comorbid anxiety and depression, for each outcome), and all three classes with persistent anxiety, depression or both were significantly associated with heart problems (highest OR for comorbid anxiety and depression). Further, high comorbid anxiety and depression symptoms was the only DAWBA domain associated with obesity, sleep problems, alcohol misuse and cannabis misuse at 24 years. Finally, in relation to individual adverse education/employment outcomes at 24 years, all three classes with persistent anxiety, depression or both were significantly associated with not being in education/employed/training (highest OR for comorbid anxiety and depression), and persistent high anxiety was the only DAWBA outcome associated with having difficulties in keeping up with work.

## Discussion

### Main findings

To our knowledge, this is the first longitudinal study to examine the associations between persistent high levels of depression, anxiety, and comorbid anxiety and depression across childhood and adolescence with several adverse outcomes at 24 years old, including mental health, physical health, substance misuse and education/employment outcomes.

### Interpretation of our findings

First, we found different patterns of development over time when we compared the trajectories from each domain (i.e. anxiety, depression, and comorbid anxiety and depression). We found trajectories suggesting that clinical symptoms of generalised anxiety (i.e. DAWBA generalised anxiety) might decrease from childhood to adolescence, whereas the clinical symptoms of depression increase. These findings are consistent with previous work, which supports that anxiety symptoms generally decline from childhood to adolescence,^15^ whereas depression becomes increasingly more prevalent across adolescence.^29^ Further, when we examined comorbid anxiety and depression over time we observed that at 8 years, children showed low levels of comorbid anxiety and depression, but at 10 and 13 years there was a clear differentiation, with a small group showing persistent and increasing high levels from 10 to 13 years.

So far, there is no previous evidence on the developmental trajectories of comorbid anxiety and depression across childhood and adolescence. The scarce existing research has focused on the broader concept of internalising symptoms, with some reporting a curvilinear trajectory where internalising symptoms decrease from age 7 to 12 years and then increase into adolescence,^14^ whereas others have revealed that child internalising symptoms increase from 4 to 10 years.^30^ As conceived by Achenbach, internalising symptoms refer more generally to problems of withdrawal, somatic complaints and anxiety/depression. Therefore, this is a broader concept than the domain of comorbid anxiety and depression included in our study, which may partially explain the discrepancies. Importantly, in each of the three domains, we detected a group of individuals characterised by high levels of symptomatology over time. This suggests potential chronicity (or persistence) of depression and anxiety, which is clinically relevant, and can develop very early in childhood and adolescence. Chronicity is not only an issue facing adults, but its potential presence in childhood is also likely to be limiting the psychological, academic and social functioning of individuals.

Second, we found that presenting with high levels of anxiety, depression and/or comorbid anxiety and depression from childhood to adolescence is critically important to psychiatry, public health and primary care, given that the persistence of symptoms was associated with a range of adverse outcomes at 24 years. We also reported some differences in the prospective associations in terms of the domains assessed (i.e. anxiety, depression, and comorbid anxiety and depression). More specifically, we found that overall, high levels of comorbid anxiety and depression was the domain that exerted the greatest negative impact at 24 years, especially on mental health and on physical health. For instance, high levels of comorbid anxiety and depression showed the greatest impact on psychotic disorder, depression, asthma, arthritis, heart problems and unemployment at 24 years, compared with persistent high levels of anxiety or depression alone. Further, high levels of comorbid anxiety and depression was the only domain associated with obesity, sleep problems and substance misuse at 24 years. This supports the notion that comorbid anxiety and depression is associated with worse overall outcomes.^10^ More specifically, comorbid anxiety and depression has been consistently associated with higher illness severity, chronicity and impairments in everyday life, in addition to more disability and alcohol problems.^31^

There are several potential reasons underlying the risk for adverse outcomes associated with adolescent depression and/or anxiety. For example, chronic elevation of stress hormones or pluripotent genomic vulnerability could lead to risk of physical and mental health conditions in the long term.^32^ Biological mechanisms such as decreased heart rate variability,^33^ autonomic nervous system dysregulation^34^ or higher inflammatory levels^35^ among individuals with depression and/or anxiety may also lead to increased risk for adverse physical (e.g. cardiovascular diseases, arthritis, diabetes) and mental health outcomes (e.g. psychosis, anxiety, depression). Further, experiencing depression and anxiety since early life might affect the social, cognitive and academic development of the child,^36^ which subsequently might increase the probabilities of presenting with adverse outcomes in adulthood. Finally, lifestyle risk factors, such as smoking, poor diet or inactivity are more frequent in those with emerging mental health disorders, which could negatively influence physical and mental health.^37^

### Strengths and limitations

Strengths of this study include the large population-based sample and the inclusion of several adverse outcomes covering a wide range of mental health, physical health and education/employment areas. There are also some limitations.

First, other potential contributing factors, such as cognition, social interactions, lifestyle and family factors, and/or obstetric complications were left unexplored. Second, we were unable to use anxiety and depression data in those older than 13 years, thus we cannot comment on future symptomatology trajectory after this age group. Third, the DAWBA measures were parent reported, thus there is a bias associated with parents’ perceptions. This parent-reported bias might be especially relevant as children get older (e.g. at 13 years old), as at these ages the parents might not be fully aware of how the child feels. Therefore, future studies should also consider self-reported and/or teacher-reported information. Fourth, the majority of participants were of White ethnicity and they were all residents in the same geographic area in the UK (Avon area), which limits the generalizability of findings to other ethnic groups and ethnic comparisons, and to other geographical areas within and outside the UK, respectively.^20^ Fifth, as is usual in birth cohort studies, the attrition rate was significant. Although we used procedures to ensure the representativeness of our results, this should be taken into account when interpreting our findings. Sixth, some of the physical health problems at 24 years included very few cases (e.g. stroke/cancer, or kidney diseases), as these are usually rare in young adulthood. Therefore, these specific results should be cautiously interpreted as these may not be representative of this specific age range. Seventh, our data-set did not include information on participants medication. Therefore, we are unaware of any medication that the participants were taking at any of the time points in this study, and which could potentially affect our findings. However, and considering that this is a population-based study, it is likely that only a very small number of participants would be on medication during the study. Finally, adjusted BIC or sample-size adjusted BIC were not used in the selection of the best-class solution, as these are not provided by our software (i.e. Mplus), but we used BIC instead. Nevertheless, there is evidence supporting BIC being an optimal information criterion.^38^

### Implications

In summary, our findings showed that depression, anxiety, and comorbid anxiety and depression from childhood to adolescence present distinct developmental patterns. For each of these domains, we detected a group of individuals characterised by persistent high levels of symptomatology. Further, high levels of anxiety, depression, and comorbid anxiety and depression from 8 to 13 years were all associated with a range of adverse outcomes at 24 years, including mental health, physical health, substance misuse and poor education/employment status.

Among these, high levels of comorbid anxiety and depression exerted the greatest adverse impact at 24 years. These findings highlight the relevance of identifying and treating those children and adolescents presenting with persistent anxiety and depression, and especially the comorbidity of both symptoms, as these would be the individuals at highest risk of having adverse outcomes at 24 years, including health and education/employment problems. Overall, our findings are important to psychiatric practice, public health and primary care, and could help early identification of those at highest risk of a range of adverse outcomes in adulthood: for example, for whom transitional services from child and adolescent mental health services into adult mental health services needs strengthening, or for whom chronicity of depression and anxiety in childhood and adolescence needs more assertive management. Our results suggest that by implementing early intervention programmes targeting chronicity of childhood depression and anxiety there is potential to have an impact on subsequent adverse health and functional outcomes in adulthood. How this could be achieved needs further research.

## Data Availability

Access to ALSPAC data is through a system of managed open access (http://www.bristol.ac.uk/alspac/researchers/access/).

## Supplementary material

For supplementary material accompanying this paper visit https://doi.org/10.1192/bjp.2023.23. click here to view supplementary material

## References

[1] Erskine HE, Moffitt TE, Copeland WE, Costello EJ, Ferrari AJ, Patton G, A heavy burden on young minds: the global burden of mental and substance use disorders in children and youth. Psychol Med 2015; 45: 1551–63. 25534496 10.1017/S0033291714002888 PMC5922255

[2] Costello EJ, Egger HL, Angold A. The developmental epidemiology of anxiety disorders: phenomenology, prevalence, and comorbidity. Child Adolesc Psychiatr Clin N Am 2005; 14: 631–48. 16171696 10.1016/j.chc.2005.06.003

[3] Puura K, Tamminen T, Almqvist F, Kresanov K, Kumpulainen K, Moilanen I, Should depression in young school-children be diagnosed with different criteria? Eur Child Adolesc Psychiatry 1997; 6: 12–9. 9112042 10.1007/BF00573635

[4] Avenevoli S, Swendsen J, He JP, Burstein M, Merikangas KR. Major depression in the national comorbidity survey–adolescent supplement: prevalence, correlates, and treatment. J Am Acad Child Adolesc Psychiatry 2015; 54: 37–44.e2. 25524788 10.1016/j.jaac.2014.10.010 PMC4408277

[5] Polanczyk G V, Salum GA, Sugaya LS, Caye A, Rohde LA. Annual research review: a meta-analysis of the worldwide prevalence of mental disorders in children and adolescents. J Child Psychol Psychiatry 2015; 56: 345–65. 25649325 10.1111/jcpp.12381

[6] Castelpietra G, Knudsen AKS, Agardh EE, Armocida B, Beghi M, Iburg KM, The burden of mental disorders, substance use disorders and self-harm among young people in Europe, 1990–2019: findings from the global burden of dsease study 2019. Lancet Reg Heal Eur 2022; 16: 100341. 10.1016/j.lanepe.2022.100341 PMC8980870 35392452

[7] Garber J, Weersing VR. Comorbidity of anxiety and depression in youth: Implications for treatment and prevention. Clin Psychol Sci Pract 2010; 17: 293–306. 10.1111/j.1468-2850.2010.01221.x PMC3074295 21499544

[8] Axelson DA, Birmaher B. Relation between anxiety and depressive disorders in childhood and adolescence. Depress Anxiety 2001; 14: 67–78. 11668659 10.1002/da.1048

[9] Fava M, Alpert JE, Carmin CN, Wisniewski SR, Trivedi MH, Biggs MM, Clinical correlates and symptom patterns of anxious depression among patients with major depressive disorder in STAR*D. Psychol Med 2004; 34: 1299–308. 15697056 10.1017/s0033291704002612

[10] Kalin NH. The critical relationship between anxiety and depression. Am J Psychiatry 2020; 177: 365–7. 32354270 10.1176/appi.ajp.2020.20030305

[11] Copeland WE, Angold A, Shanahan L, Costello EJ. Longitudinal patterns of anxiety from childhood to adulthood: the Great Smoky Mountains Study. J Am Acad Child Adolesc Psychiatry 2014; 53: 21–33. 24342383 10.1016/j.jaac.2013.09.017 PMC3939681

[12] Glied S, Pine DS. Consequences and correlates of adolescent depression. Arch Pediatr Adolesc Med 2002; 156: 1009. 12361447 10.1001/archpedi.156.10.1009

[13] Copeland WE, Alaie I, Jonsson U, Shanahan L. Associations of childhood and adolescent depression with adult psychiatric and functional outcomes. J Am Acad Child Adolesc Psychiatry 2021; 60: 604–11. 32758528 10.1016/j.jaac.2020.07.895 PMC8051642

[14] Cohen JR, Andrews AR, Davis MM, Rudolph KD. Anxiety and depression during childhood and adolescence: testing theoretical models of continuity and discontinuity. J Abnorm Child Psychol 2018; 46: 1295–308. 29256025 10.1007/s10802-017-0370-x PMC6008170

[15] Allan NP, Capron DW, Lejuez CW, Reynolds EK, MacPherson L, Schmidt NB. Developmental trajectories of anxiety symptoms in early adolescence: the influence of anxiety sensitivity. J Abnorm Child Psychol 2014; 42: 589–600. 24062146 10.1007/s10802-013-9806-0 PMC4046901

[16] Dekker MC, Ferdinand RF, van Lang NDJ, Bongers IL, van der Ende J, Verhulst FC. Developmental trajectories of depressive symptoms from early childhood to late adolescence: gender differences and adult outcome. J Child Psychol Psychiatry 2007; 48: 657–66. 17593146 10.1111/j.1469-7610.2007.01742.x

[17] McLaughlin KA, King K. Developmental trajectories of anxiety and depression in early adolescence. J Abnorm Child Psychol 2015; 43: 311–23. 24996791 10.1007/s10802-014-9898-1 PMC4286282

[18] Prinzie P, van Harten LV, Deković M, van den Akker AL, Shiner RL. Developmental trajectories of anxious and depressive problems during the transition from childhood to adolescence: personality × parenting interactions. Dev Psychopathol 2014; 26: 1077–92. 24914625 10.1017/S0954579414000510

[19] López-López JA, Kwong ASF, Washbrook E, Pearson RM, Tilling K, Fazel MS, Trajectories of depressive symptoms and adult educational and employment outcomes. BJPsych Open 2020; 6: e6. 10.1192/bjo.2019.90 PMC7001468 31829293

[20] Fraser A, Macdonald-Wallis C, Tilling K, Boyd A, Golding J, Smith GD, Cohort profile: the Avon Longitudinal Study of Parents and Children: ALSPAC mothers cohort. Int J Epidemiol 2013; 42: 97–110. 22507742 10.1093/ije/dys066 PMC3600619

[21] Goodman R, Ford T, Richards H, Gatward R, Meltzer H. The Development and Well-Being Assessment: description and initial validation of an integrated assessment of child and adolescent psychopathology. J Child Psychol Psychiatry 2000; 41: 645–55. 10946756

[22] Zammit S, Horwood J, Thompson A, Thomas K, Menezes P, Gunnell D, Investigating if psychosis-like symptoms (PLIKS) are associated with family history of schizophrenia or paternal age in the ALSPAC birth cohort. Schizophr Res 2008; 104: 279–86. 18562177 10.1016/j.schres.2008.04.036

[23] Angst J, Adolfsson R, Benazzi F, Gamma A, Hantouche E, Meyer TD, The HCL-32: towards a self-assessment tool for hypomanic symptoms in outpatients. J Affect Disord 2005; 88: 217–33. 16125784 10.1016/j.jad.2005.05.011

[24] Brugha TS, Bebbington PE, Jenkins R, Meltzer H, Taub NA, Janas M, Cross validation of a general population survey diagnostic interview: a comparison of CIS-R with SCAN ICD-10 diagnostic categories. Psychol Med 1999; 29: 1029–42. 10576296 10.1017/s0033291799008892

[25] Kivimäki M, Strandberg T, Pentti J, Nyberg ST, Frank P, Jokela M, Body-mass index and risk of obesity-related complex multimorbidity: an observational multicohort study. Lancet Diabetes Endocrinol 2022; 10(4): 253–63. 35248171 10.1016/S2213-8587(22)00033-X PMC8938400

[26] American Psychiatric Association. Diagnostic and Statistical Manual of Mental Disorders *(4th edn)*. American Psychiatric Association, 1994.

[27] Legleye S, Karila L, Beck F, Reynaud M. Validation of the CAST, a general population Cannabis abuse screening test. J Subst Use 2007; 12: 233–42.

[28] Wiggins JL, Mitchell C, Hyde LW, Monk CS. Identifying early pathways of risk and resilience: the codevelopment of internalizing and externalizing symptoms and the role of harsh parenting. Dev Psychopathol 2015; 27: 1295–312. 26439075 10.1017/S0954579414001412 PMC4961476

[29] Hankin BL. Depression from childhood through adolescence: risk mechanisms across multiple systems and levels of analysis. Curr Opin Psychol 2015; 4: 13–20. 25692174 10.1016/j.copsyc.2015.01.003 PMC4327904

[30] Shanahan L, Calkins SD, Keane SP, Kelleher R, Suffness R. Trajectories of internalizing symptoms across childhood: the roles of biological self-regulation and maternal psychopathology. Dev Psychopathol 2014; 26: 1353–68. 25422966 10.1017/S0954579414001072 PMC4678417

[31] Belzer K, Schneier FR. Comorbidity of anxiety and depressive disorders: issues in conceptualization, assessment, and treatment. J Psychiatr Pract 2004; 10: 296–306. 15361744 10.1097/00131746-200409000-00003

[32] De Deurwaerdère P, Di Giovanni G. Serotonin in health and disease. Int J Mol Sci 2020; 21: 3500. 10.3390/ijms21103500 PMC7278959 32429111

[33] Jung W, Jang KI, Lee SH. Heart and brain interaction of psychiatric illness: a review focused on heart rate variability, cognitive function, and quantitative electroencephalography. Clin Psychopharmacol Neurosci 2019; 17: 459–74. 31671483 10.9758/cpn.2019.17.4.459 PMC6852682

[34] Van Lieshout RJ, Bienenstock J, MacQueen GM. A review of candidate pathways underlying the association between asthma and major depressive disorder. Psychosom Med 2009; 71: 187–95. 19073754 10.1097/PSY.0b013e3181907012

[35] Miller GE, Stetler CA, Carney RM, Freedland KE, Banks WA. Clinical depression and inflammatory risk markers for coronary heart disease. Am J Cardiol 2002; 90: 1279–83. 12480034 10.1016/s0002-9149(02)02863-1

[36] Jaycox LH, Stein BD, Paddock S, Miles JNV, Chandra A, Meredith LS, Impact of teen depression on academic, social, and physical functioning. Pediatrics 2009; 124: e596–605. 19736259 10.1542/peds.2008-3348

[37] Firth J, Siddiqi N, Koyanagi A, Siskind D, Rosenbaum S, Galletly C, The *Lancet Psychiatry* Commission: a blueprint for protecting physical health in people with mental illness. Lancet Psychiatry 2019; 6: 675–712. 31324560 10.1016/S2215-0366(19)30132-4

[38] Nylund KL, Asparouhov T, Muthén BO. Deciding on the number of classes in latent class analysis and growth mixture modeling: a mnte carlo simulation study. Struct Equ Model A Multidiscip J 2007; 14: 535–69.

