# Supplementary material for: Impact of anxiety and depression across childhood and adolescence on adverse outcomes in young adulthood: a UK birth cohort study
Source: Br J Psychiatry. 2023 May;222(5):212–20. doi: 10.1192/bjp.2023.23 (PMC10895507; doi:10.1192/bjp.2023.23)
Supplement: Supplementary file 1 [file S0007125023000235sup001.docx]

**Supplementary material: online-only document**

1. Supplementary Appendix 1: Further details of ALSPAC cohort (page 2)
2. Supplementary Appendix 2: Further details of the DAWBA (page 3)
3. Table S1. Items comprising the DAWBA generalized anxiety and DAWBA mood (page 4)
4. Table S2. Description of the measures used to capture mental health at 24 years (page 5)
5. Table S3. DSM-IV-TR Diagnostic Criteria for Alcohol Abuse (page 6)
6. Table S4. Differences in socio-demographic variables between non-participating and participating subjects in the study (page 7)
7. Table S5. Unadjusted logistic regressions between LCGA 3-classes model and adverse outcomes at 24 years (page 8)
8. Table S6a. Associations between LCGA and mental health problems at 24 years (page 9)
9. Table S6b. Associations between LCGA and mental health problems at 24 years (page 10)
10. Table S6c. Associations between LCGA and mental health problems at 24 years (page 11)
11. Table S7a. Associations between LCGA and physical health problems at 24 years (page 12)
12. Table S7b. Associations between LCGA and physical health problems at 24 years (page 13)
13. Table S7c. Associations between LCGA and physical health problems at 24 years (page 14)
14. Table S7d. Associations between LCGA and physical health problems at 24 years (page 15)
15. Table S8. Associations between LCGA and sleep problems at 24 years (page 16)
16. Table S9. Associations between LCGA and functional outcomes at 24 years (page 17)
17. Table S10. Associations between LCGA classes and individual substance abuse outcomes at 24 years (apge 18)

**Supplementary Appendix 2**

**Further details of the ALSPAC cohort:**

The initial number of pregnancies enrolled was 14,541 (for these at least one questionnaire was returned, or a “Children in Focus” clinic had been attended by 19/07/99). Of these initial pregnancies, there was a total of 14,676 foetuses, resulting in 14,062 live births and 13,988 children who were alive at 1 year of age. When the oldest children were approximately 7 years of age, an attempt was made to bolster the initial sample with eligible cases who had failed to join the study originally. As a result, in our study, as some variables were collected from the age of seven onwards there were data available for more than the 14,541 pregnancies mentioned above. Informed consent for the use of data collected via questionnaires and clinics was obtained from participants following the recommendations of the ALSPAC Ethics and Law Committee at the time. Ethical approval was obtained from the ALSPAC Ethics and Law Committee and the local research ethics committees. Regarding the recruitment strategies used, the substantial majority (82.6%) of women are known to have been invited to enrol during the 1990–92 recruitment campaign. With funding to complete a ‘Focus@7’ follow-up assessment of all of the participants at 7 years, the opportunity was taken to attempt to recruit all known eligible children who would have fitted the original eligibility criteria. Invitations describing the study were sent to this group, inviting the mothers to enrol. In addition to this systematic recruitment, ALSPAC made subsequent opportunistic contact with additional families who were ‘eligible’ but not ‘enrolled’. Contact was initiated by eligible families seeking enrolment or during ALSPAC community outreach and promotion activities. Concerning data collection, assessments have been administered frequently, with more than 68 data collection time points between birth and 24 years of age. Additional follow-up of the ‘eligible sample’ has been made through school-administered questionnaires and assessments completed by the child’s teacher. The ‘eligible sample’ has been also linked to National Health Service death and cancer registries and education attainment and school census data. Regarding attrition, attrition rates were at their greatest when the child was in infancy and are increasing again as the children enter adulthood.

**Supplementary Appendix 2**

**Further details of the DAWBA:**

The DAWBA is a package of interviews, questionnaires and rating techniques designed to generate ICD-10 and DSM-IV or DSM-5 psychiatric diagnoses about 2-17 years old. The DAWBA includes a mix of ‘closed’/structured questions and open-ended questions, where respondents describe their difficulties in their own words. The full DAWBA package covers the following diagnoses: Separation anxiety, Specific phobia, Social phobia, Panic disorder/agoraphobia, Post-traumatic stress disorder, Obsessive compulsive disorder, Generalized anxiety disorder, Body dysmorphic disorder, Disruptive mood dysregulation disorder, Major depression, ADHD/hyperkinesis, Oppositional defiant disorder, Conduct disorder, Eating disorders, including anorexia, bulimia and binge eating, Autism spectrum disorders, Tic disorders, including Tourette syndrome, and Bipolar disorders. For each of these disorders, the interview asks about all the symptoms, and other criteria needed for an operationalized diagnosis according to both DSM-IV (American Psychiatric Association, 1994) and the research diagnostic version of ICD-10 (World Health Organisation, 1994). Panic disorder, agoraphobia, autistic disorders, eating disorders, tic disorders, and any other concerns are covered more briefly, with clinical diagnoses of these disorders being correspondingly more dependent on rating the open-ended transcript. The time frame of the interview is the present and the recent past. For many disorders, the ICD-10 and DSM-IV diagnostic criteria stipulate that the symptoms need to have persisted for a specified number of months, e.g. a minimum of 6 months for hyperactivity, oppositional-defiant disorder, and generalized anxiety disorders. In these instances, the relevant section of the DAWBA interview focuses on the child’s symptoms over this stipulated period. The time frame is longest for conduct disorder (since DSM-IV criteria include the number of relevant behaviours displayed over the previous 12 months), and shortest for most of the emotional disorders, where the focus is on the last month, in line with previous recommendations (Shaffer et al., 1996).

**Table S1. Description of the items for the DAWBA generalized anxiety and the DAWBA mood**

| **General Anxieties Score** |
| --- |
| 1. Child worries about past behavior.      1. Child worries about school-work.      1. Child worries about disasters.      1. Child worries about own health.      1. Child worries about bad things happening to others. 2. Child worries about the future.      1. Child worries about other things. |
| **General Anxieties Symptoms Score** |
| 1. Worries lead to child being restless, tense or on edge. 2. Worries lead to child being easily tired. 3. Worries lead to difficulties in concentrating. 4. Worries lead to irritability.      1. Worries lead to child looking tense.      1. Worries interfere with sleep. |
| **Mood total score** |
| 1. Child has been miserable/irritable/lacked interest in things usually enjoys in past month 2. Child had no energy/seemed tired all the time in past month      1. Child was eating much more/less than usual in past month      1. Child lost/gained a lot of weight in past month      1. Child had difficulty getting to sleep in past month      1. Child slept too much in past month      1. Child thought about death a lot in past month      1. Child ever talked about harming/killing them self in past month      1. Child ever tried to harm/kill them self in past month      1. Child ever tried to harm/kill them self during their lifetime |

| **Psychotic disorder at 24 years** |
| --- |
| Psychotic experiences (PEs) were identified through the semi-structured Psychosis-Like Symptom Interview. Psychotic experiences covered the three main positive symptom domains: hallucinations, delusions, and thought interference. Interviewers rated psychotic experiences as not present, suspected, or definitely present. We identified Psychotic disorder at 24 years as the outcome, which was defined as 1) being rated as having definite PEs not attributable to the effects of sleep or fever; 2) having recurred regularly (at least once per month) over the previous 6 months; and 3) being reported as either very distressing or having a very negative impact on their social or occupational functioning or led them to seek help from a professional source. |
| **Hypomania at 22-23 years** |
| Hypomania symptoms were defined using the Hypomania Checklist, a self-report measure of lifetime experience of manic symptoms. Participants were asked to consider a time when they were in a “high or hyper” state and to endorse a number of statements about their emotions, thoughts, and behaviors at that time. We defined lifetime history of hypomania as follows: a score of 14 or more out of 32 hypomanic features; plus at least one response of either “negative consequences” or “negative plus positive consequences”; plus a report that these mood changes caused a reaction in others; plus a duration of “2–3 days” or more. To note, the Hypomania Checklist was only used when the young person was 22-23 years, and not at 24 years old. Therefore, this was the only mental health outcome which was collected at 22-23 years, instead of at 24 years. |
| **Severe depression, GAD, Social phobia, Specific phobia and Panic disorder at 24 years** |
| The CIS-R is a structured interview examining the presence of symptoms of common mental disorders in the past week. It covers 14 types of common mental disorder symptoms (somatic symptoms, fatigue, concentration and forgetfulness, depression, depressive ideas, worry, anxiety, sleep problems, irritability, worry about physical health, phobias, panic, compulsions and obsessions), and six (non-mutually exclusive) ICD-10 disorders (Generalized anxiety disorder, depression, phobias, obsessive compulsive disorder, panic disorder, and common mental disorder not otherwise specified [NOS]), together with a continuous scale that reflects the overall severity of common mental disorder psychopathology. The CIS-R has been shown to be equally reliable when administered by interviewer or in a computer-assisted self-administered format. It has been widely used in population surveys. |

**Table S2. Description of the measures used to capture mental health problems at 24 years**

**Table S3. DSM-IV-TR Diagnostic Criteria for Alcohol Abuse**

| **Alcohol abuse at 24 years** |
| --- |
| (A) A maladaptive pattern of drinking, leading to clinically significant impairment or distress, as manifested by at least one of the following occurring within a 12-month period:  Recurrent use of alcohol resulting in a failure to fulfill major role obligations at work, school, or home (e.g., repeated absences or poor work performance related to alcohol use; alcohol-related absences, suspensions, or expulsions from school; neglect of children or household)  Recurrent alcohol use in situations in which it is physically hazardous (e.g., driving an automobile or operating a machine when impaired by alcohol use)  Recurrent alcohol-related legal problems (e.g., arrests for alcohol-related disorderly conduct)  Continued alcohol use despite having persistent or recurrent social or interpersonal problems caused or exacerbated by the effects of alcohol (e.g., arguments with spouse about consequences of intoxication).  (B) Never met criteria for alcohol dependence. |

**Table S4.** Differences in socio-demographic variables between non-participating and participating subjects in the study

|  | **Non-participating group in the study** | | **Participating group in the study (at 24 years old)** | | | **Non-participating versus participating** | |
| --- | --- | --- | --- | --- | --- | --- | --- |
|  | *Mean* | *SD* | *Mean* | *SD* | | *OR (95% CI)* | *p* |
| Maternal age when born | 27.49 | 4.99 | 29.45 | 4.56 | | 1.08 (1.07, 1.09) | <0.001 |
| Gestational age | 38.00 | 6.22 | 39.49 | 1.80 | | 1.09 (1.08, 1.11) | <0.001 |
| Birth weight, kg | 3371.23 | 596.18 | 3410.33 | 532.74 | | 1.25 (1.05, 1.20) | <0.001 |
| Family Adversity score | 4.74 | 4.47 | 3.61 | 3.84 | | 0.94 (0.93, 0.95) | <0.001 |
|  | **Non-participating group in the study** | | **Participating group in the study** | | |  |  |
|  | *N* | *%* | *N* | | *%* |  |  |
| Sex  Male / Female | 6233 / 4919 | 55.9 / 44.1 | 1458 / 2429 | | 37.5 / 62.5 | 0.47 (0.44, 0.51) | <0.001 |
| Ethnicity |  |  |  | |  |  |  |
| White / Other | 8657 / 248 | 97.2 / 2.8 | 3405 / 78 | | 97.8 / 2.2 | 1.25 (0.97, 1.62) | 0.089 |

The individuals associated with attrition at 24 years were more often boys, their mothers were younger when baby was born, the gestational age was shorter, they weighted less at birth, and they had higher socioeconomic levels.

| **SUBSTANCE ABUSE AT 24 YEARS** | | | | | | | | | | | |
| --- | --- | --- | --- | --- | --- | --- | --- | --- | --- | --- | --- |
| **DAWBA ANXIETY** | | | | **DAWBA DEPRESSION** | | | | **DAWBA ANXIETY + DEPRESSION** | | | |
|  | OR | 95% CI | p |  | OR | 95% CI | p |  | OR | 95% CI | p |
| Anxiety Class1 (ref) | --- | --- | --- | Mood Class2 (ref) | --- | --- | --- | A+D Class2 (ref) | --- | --- | --- |
| Anxiety Class2 | 1.04 | 0.90 to 1.20 | 0.566 | Mood Class1 | **1.46** | **1.22 to 1.75** | **<0.001** | A+D Class1 | **1.39** | **1.03 to 1.89** | **0.033** |
| Anxiety Class3 | 0.96 | 0.73 to 1.26 | 0.764 | Mood Class3 | 1.25 | 0.93 to 1.69 | 0.144 | A+D Class3 | **1.44** | **1.20 to 1.72** | **<0.001** |
| **Any FUNCTIONING PROBLEM at 24 years** | | | | | | | | | | | |
| Anxiety Class1 (ref) | **---** | **---** | --- | Mood Class2 (ref) | **---** | **---** | --- | A+D Class2 (ref) | **---** | **---** | --- |
| Anxiety Class2 | **1.29** | **1.11 to 1.49** | **<0.001** | Mood Class1 | **1.81** | **1.51 to 2.17** | **<0.001** | A+D Class1 | **1.73** | **1.44 to 2.07** | **<0.001** |
| Anxiety Class3 | **1.68** | **1.31 to 2.17** | **<0.001** | Mood Class3 | **1.50** | **1.09 to 2.06** | **0.012** | A+D Class3 | **1.63** | **1.19 to 2.25** | **0.003** |

**Table 5.** Unadjusted logistic regressions between LCGA 3-classes model and adverse outcomes at 24 years

| **Any MENTAL HEALTH PROBLEM at 24 years** | | | | | | | | | | | |
| --- | --- | --- | --- | --- | --- | --- | --- | --- | --- | --- | --- |
| **DAWBA ANXIETY** | | | | **DAWBA DEPRESSION** | | | | **DAWBA ANXIETY + DEPRESSION** | | | |
|  | OR | 95% CI | p |  | OR | 95% CI | p |  | OR | 95% CI | p |
| Anxiety Class1 (ref) | --- | --- | --- | Mood Class2 (ref) | --- | --- | --- | A+D Class2 (ref) | --- | --- | --- |
| Anxiety Class2 | 1.17 | 0.99 to 1.36 | 0.051 | Mood Class1 | **1.35** | **1.10 to 1.65** | **0.004** | A+D Class1 | **1.32** | **1.08 to 1.61** | **0.007** |
| Anxiety Class3 | **1.56** | **1.20 to 2.04** | **0.001** | Mood Class3 | **1.85** | **1.38 to 2.49** | **<0.001** | A+D Class3 | **1.71** | **1.26 to 2.33** | **<0.001** |
| **Any PHYSICAL HEALTH PROBLEM at 24 years** | | | | | | | | | | | |
| Anxiety Class1 (ref) | --- | --- | --- | Mood Class2 (ref) | --- | --- | --- | A+D Class2 (ref) | **---** | **---** | --- |
| Anxiety Class2 | 1.21 | 1.05 to 1.39 | 0.008 | Mood Class1 | **1.31** | **1.09 to 1.57** | **0.004** | A+D Class1 | **1.26** | **1.04 to 1.52** | **0.018** |
| Anxiety Class3 | 1.17 | 0.90 to 1.53 | 0.245 | Mood Class3 | **1.60** | **1.20 to 2.14** | **0.002** | A+D Class3 | **2.08** | **1.60 to 2.71** | **<0.001** |

**Table S6a.** Associations between LCGA classes and individual mental health problems at 24 years

| **Psychotic disorder at 24 years** | | | | | | | | | | | |
| --- | --- | --- | --- | --- | --- | --- | --- | --- | --- | --- | --- |
| **DAWBA ANXIETY** | | | | **DAWBA DEPRESSION** | | | | **DAWBA ANXIETY + DEPRESSION** | | | |
|  | OR | 95% CI | p |  | OR | 95% CI | p |  | OR | 95% CI | p |
| Anxiety Class1 (ref) | --- | --- | --- | Mood Class2 (ref) | --- | --- | --- | A+D Class2 (ref) | --- | --- | --- |
| Anxiety Class2 | 1.06 | 0.67 to 1.67 | 0.808 | Mood Class1 | 1.03 | 0.55 to 1.93 | 0.918 | A+D Class1 | 1.04 | 0.56 to 1.94 | 0.893 |
| Anxiety Class3 | **2.70** | **1.50 to 4.86** | **0.001** | Mood Class3 | **3.51** | **2.01 to 6.12** | **<0.001** | A+D Class3 | **3.85** | **2.20 to 6.73** | **<0.001** |
| Sex | **0.48** | **0.32 to 0.71** | **<0.001** | Sex | **0.46** | **0.31 to 0.69** | **<0.001** | Sex | **0.46** | **0.31 to 0.68** | **<0.001** |
| Gestational age | 0.93 | 0.84 to 1.02 | 0.131 | Gestational age | **0.91** | **0.83 to 0.99** | **0.048** | Gestational age | 0.92 | 0.84 to 1.01 | 0.070 |
| FAI total score | **1.06** | **1.03 to 1.10** | **<0.001** | FAI total score | **1.06** | **1.03 to 1.10** | **<0.001** | FAI total score | **1.06** | **1.02 to 1.09** | **0.001** |
| Ethnicity | 1.18 | 0.36 to 3.88 | 0.788 | Ethnicity | 1.21 | 0.37 to 3.99 | 0.754 | Ethnicity | 1.25 | 0.38 to 4.11 | 0.717 |
| Maternal age birth | 0.97 | 0.93 to 1.01 | 0.106 | Maternal age birth | 0.97 | 0.93 to 1.01 | 0.120 | Maternal age birth | 0.98 | 0.94 to 1.02 | 0.251 |
| **Hypomania at 22-23 years** | | | | | | | | | | | |
| Anxiety Class1 (ref) | --- | --- | --- | Mood Class2 (ref) | --- | --- | --- | A+D Class2 (ref) | --- | --- | --- |
| Anxiety Class2 | 1.04 | 0.93 to 1.16 | 0.472 | Mood Class1 | 1.02 | 0.87 to 1.21 | 0.788 | A+D Class1 | 1.05 | 0.89 to 1.23 | 0.586 |
| Anxiety Class3 | 1.20 | 0.95 to 1.52 | 0.125 | Mood Class3 | 1.20 | 0.93 to 1.56 | 0.166 | A+D Class3 | 1.21 | 0.92 to 1.60 | 0.176 |
| Sex | **1.18** | **1.07 to 1.30** | **0.001** | Sex | **1.20** | **1.09 to 1.32** | **<0.001** | Sex | **1.22** | **1.11 to 1.34** | **<0.001** |
| Gestational age | 0.99 | 0.96 to 1.02 | 0.381 | Gestational age | 1.00 | 0.97 to 1.03 | 0.869 | Gestational age | 1.01 | 0.98 to 1.04 | 0.435 |
| FAI total score | 1.00 | 0.99 to 1.01 | 0.954 | FAI total score | 1.00 | 0.99 to 1.02 | 0.498 | FAI total score | 1.00 | 0.99 to 1.01 | 0.680 |
| Ethnicity | 1.06 | 0.75 to 1.51 | 0.738 | Ethnicity | 0.97 | 0.70 to 1.35 | 0.874 | Ethnicity | 0.93 | 0.68 to 1.28 | 0.668 |
| Maternal age birth | **1.01** | **1.00 to 1.02** | **0.017** | Maternal age birth | **1.02** | **1.01 to 1.03** | **0.002** | Maternal age birth | **1.01** | **1.00 to 1.02** | **0.018** |
|  |  |  |  | **Severe depression at 24 years** | | | |  |  |  |  |
| Anxiety Class1 (ref) | --- | --- | --- | Mood Class2 (ref) | --- | --- | --- | A+D Class2 (ref) | --- | --- | --- |
| Anxiety Class2 | 1.11 | 0.70 to 1.76 | 0.651 | Mood Class1 | 0.71 | 0.36 to 1.40 | 0.320 | A+D Class1 | .74 | 0.37 to 1.46 | 0.382 |
| Anxiety Class3 | **3.91** | **2.31 to 6.64** | **<0.001** | Mood Class3 | **3.80** | **2.31 to 6.25** | **<0.001** | A+D Class3 | **4.16** | **2.52 to 6.86** | **<0.001** |
| Sex | **0.52** | **0.35 to 0.77** | **0.001** | Sex | **0.52** | **0.36 to 0.76** | **0.001** | Sex | **0.52** | **0.36 to 0.77** | **0.001** |
| Gestational age | 1.04 | 0.93 to 1.17 | 0.446 | Gestational age | 1.08 | 0.96 to 1.20 | 0.190 | Gestational age | 1.09 | 0.97 to 1.21 | 0.138 |
| FAI total score | **1.12** | **1.08 to 1.15** | **<0.001** | FAI total score | **1.12** | **1.09 to 1.16** | **<0.001** | FAI total score | **1.12** | **1.09 to 1.15** | **<0.001** |
| Ethnicity | 1.18 | 0.37 to 3.76 | 0.777 | Ethnicity | 1.13 | 0.36 to 3.60 | 0.834 | Ethnicity | 1.19 | 0.37 to 3.78 | 0.769 |
| Maternal age birth | 1.04 | 0.99 to 1.08 | 0.060 | Maternal age birth | 1.02 | 0.99 to 1.06 | 0.198 | Maternal age birth | 1.03 | 0.99 to 1.06 | 0.136 |

**Table S6b.** Associations between LCGA classes and individual mental health problems at 24 years

| **Generalized anxiety disorder at 24 years** | | | | | | | | | | | |
| --- | --- | --- | --- | --- | --- | --- | --- | --- | --- | --- | --- |
| **DAWBA ANXIETY** | | | | **DAWBA DEPRESSION** | | | | **DAWBA ANXIETY + DEPRESSION** | | | |
|  | OR | 95% CI | p |  | OR | 95% CI | p |  | OR | 95% CI | p |
| Anxiety Class1 (ref) | --- | --- | --- | Mood Class2 (ref) | --- | --- | --- | A+D Class2 (ref) | **---** | **---** | --- |
| Anxiety Class2 | 1.18 | 0.99 to 1.39 | 0.063 | Mood Class1 | 1.16 | 0.93 to 1.45 | 0.189 | A+D Class1 | **1.26** | **1.01 to 1.56** | **0.037** |
| Anxiety Class3 | **2.28** | **1.76 to 2.97** | **<0.001** | Mood Class3 | **2.39** | **1.81 to 3.16** | **<0.001** | A+D Class3 | **2.08** | **1.54 to 2.81** | **<0.001** |
| Sex | **0.46** | **0.40 to 0.54** | **<0.001** | Sex | **0.51** | **0.44 to 0.59** | **<0.001** | Sex | **0.50** | **0.43 to 0.58** | **<0.001** |
| Gestational age | 0.96 | 0.93 to 1.00 | 0.070 | Gestational age | 0.97 | 0.93 to 1.01 | 0.124 | Gestational age | 0.98 | 0.94 to 1.02 | 0.310 |
| FAI total score | **1.04** | **1.03 to 1.06** | **<0.001** | FAI total score | **1.04** | **1.03 to 1.06** | **<0.001** | FAI total score | **1.04** | **1.03 to 1.06** | **<0.001** |
| Ethnicity | 0.88 | 0.51 to 1.51 | 0.645 | Ethnicity | 0.81 | 0.47 to 1.39 | 0.452 | Ethnicity | 0.81 | 0.47 to 1.39 | 0.449 |
| Maternal age birth | 0.99 | 0.98 to 1.01 | 0.770 | Maternal age birth | 0.99 | 0.98 to 1.01 | 0.646 | Maternal age birth | 0.99 | 0.98 to 1.01 | 0.698 |
| **Specific phobia at 24 years** | | | | | | | | | | | |
| Anxiety Class1 (ref) | --- | --- | --- | Mood Class2 (ref) | --- | --- | --- | A+D Class2 (ref) | --- | --- | --- |
| Anxiety Class2 | 1.58 | 0.54 to 4.63 | 0.404 | Mood Class1 | 1.61 | 0.41 to 6.24 | 0.492 | A+D Class1 | 1.62 | 0.42 to 6.26 | 0.484 |
| Anxiety Class3 | 0.00 | 0.00 to 0.00 | 0.993 | Mood Class3 | 0.00 | 0.00 to 0.00 | 0.994 | A+D Class3 | 0.00 | 0.00 to 0.00 | 0.995 |
| Sex | 0.00 | 0.00 to 0.00 | 0.978 | Sex | 0.00 | 0.00 to 0.00 | 0.978 | Sex | 0.00 | 0.00 to 0.00 | 0.978 |
| Gestational age | 1.14 | 0.80 to 1.62 | 0.465 | Gestational age | 1.13 | 0.81 to 1.59 | 0.473 | Gestational age | 1.14 | 0.80 to 1.62 | 0.465 |
| FAI total score | 0.96 | 0.84 to 1.09 | 0.522 | FAI total score | 0.96 | 0.85 to 1.09 | 0.543 | FAI total score | 0.96 | 0.84 to 1.09 | 0.522 |
| Ethnicity | **9.09** | **2.15 to 38.48** | **0.003** | Ethnicity | **8.59** | **2.04 to 36.17** | **0.003** | Ethnicity | **8.75** | **2.15 to 38.48** | **0.003** |
| Maternal age birth | 0.93 | 0.82 to 1.04 | 0.209 | Maternal age birth | 0.93 | 0.83 to 1.05 | 0.225 | Maternal age birth | 0.93 | 0.82 to 1.04 | 0.209 |
|  |  |  |  | **Social phobia at 24 years** | | | |  |  |  |  |
| Anxiety Class1 (ref) | --- | --- | --- | Mood Class2 (ref) | --- | --- | --- | A+D Class2 (ref) | --- | --- | --- |
| Anxiety Class2 | 0.83 | 0.27 to 2.61 | 0.756 | Mood Class1 | 0.00 | 0.00 to 0.00 | 0.991 | A+D Class1 | 0.00 | 0.00 to 0.00 | 0.990 |
| Anxiety Class3 | 0.00 | 0.00 to 0.00 | 0.993 | Mood Class3 | 0.00 | 0.00 to 0.00 | 0.994 | A+D Class3 | 0.00 | 0.00 to 0.00 | 0.994 |
| Sex | 0.47 | 1.67 to 1.31 | 0.151 | Sex | 0.45 | 0.16 to 1.26 | 0.129 | Sex | 0.47 | 1.67 to 1.31 | 0.151 |
| Gestational age | 1.24 | 0.88 to 1.75 | 0.219 | Gestational age | 1.19 | 0.86 to 1.64 | 0.289 | Gestational age | 1.24 | 0.88 to 1.75 | 0.219 |
| FAI total score | **1.13** | **1.06 to 1.21** | **<0.001** | FAI total score | **1.14** | **1.06 to 1.22** | **<0.001** | FAI total score | **1.13** | **1.06 to 1.21** | **<0.001** |
| Ethnicity | 0.00 | 0.00 to 0.00 | 0.996 | Ethnicity | 0.00 | 0.00 to 0.00 | 0.996 | Ethnicity | 0.00 | 0.00 to 0.00 | 0.996 |
| Maternal age birth | 1.01 | 0.92 to 1.11 | 0.863 | Maternal age birth | 1.01 | 0.92 to 1.11 | 0.766 | Maternal age birth | 1.01 | 0.92 to 1.11 | 0.863 |

**Table S6c.** Associations between LCGA classes and individual mental health problems at 24 years

| **Panic disorder at 24 years** | | | | | | | | | | | |
| --- | --- | --- | --- | --- | --- | --- | --- | --- | --- | --- | --- |
| **DAWBA ANXIETY** | | | | **DAWBA DEPRESSION** | | | | **DAWBA ANXIETY + DEPRESSION** | | | |
|  | OR | 95% CI | p |  | OR | 95% CI | p |  | OR | 95% CI | p |
| Anxiety Class1 (ref) | --- | --- | --- | Mood Class2 (ref) | --- | --- | --- | A+D Class2 (ref) | --- | --- | --- |
| Anxiety Class2 | 1.25 | 0.73 to 2.15 | 0.422 | Mood Class1 | 0.61 | 0.24 to 1.53 | 0.294 | A+D Class1 | 0.60 | 0.24 to 1.52 | 0.282 |
| Anxiety Class3 | **3.19** | **1.60 to 6.34** | **0.001** | Mood Class3 | **2.93** | **1.44 to 5.95** | **0.003** | A+D Class3 | **3.15** | **1.55 to 6.42** | **0.002** |
| Sex | **0.18** | **0.10 to 0.33** | **<0.001** | Sex | **0.18** | **0.10 to 0.34** | **<0.001** | Sex | **0.18** | **0.10 to 0.33** | **<0.001** |
| Gestational age | **1.23** | **1.05 to 1.45** | **0.010** | Gestational age | **1.22** | **1.04 to 1.42** | **0.013** | Gestational age | **1.22** | **1.05 to 1.43** | **0.010** |
| FAI total score | 1.04 | 0.99 to 1.08 | 0.108 | FAI total score | 1.04 | 0.99 to 1.08 | 0.104 | FAI total score | 1.03 | 0.99 to 1.08 | 0.179 |
| Ethnicity | 2.16 | 0.65 to 7.19 | 0.210 | Ethnicity | 2.17 | 0.65 to 7.24 | 0.207 | Ethnicity | 2.17 | 0.65 to 7.24 | 0.207 |
| Maternal age birth | 0.99 | 0.94 to 1.04 | 0.699 | Maternal age birth | 1.00 | 0.96 to 1.05 | 0.864 | Maternal age birth | 1.01 | 0.97 to 1.06 | 0.528 |

**Table S7a.** Associations between LCGA classes and individual physical health problems at 24 years

| **DIABETES at 24 years** | | | | | | | | | | | |
| --- | --- | --- | --- | --- | --- | --- | --- | --- | --- | --- | --- |
| **DAWBA ANXIETY** | | | | **DAWBA DEPRESSION** | | | | **DAWBA ANXIETY + DEPRESSION** | | | |
|  | OR | 95% CI | p |  | OR | 95% CI | p |  | OR | 95% CI | p |
| Anxiety Class1 (ref) | --- | --- | --- | Mood Class2 (ref) | --- | --- | --- | A+D Class2 (ref) | --- | --- | --- |
| Anxiety Class2 | .49 | .12 to 1.97 | .315 | Mood Class1 | .00 | .00 to .00 | .992 | A+D Class1 | .00 | .00 to .00 | .992 |
| Anxiety Class3 | **3.65** | **1.15 to 11.64** | **.028** | Mood Class3 | .00 | .00 to .00 | .995 | A+D Class3 | .00 | .00 to .00 | .995 |
| Sex | .45 | .17 to 1.17 | .103 | Sex | .45 | .17 to 1.17 | .096 | Sex | .44 | .17 to 1.16 | .097 |
| Gestational age | .97 | .75 to 1.26 | .847 | Gestational age | 1.00 | .76 to 1.31 | .991 | Gestational age | .99 | .76 to 1.31 | .986 |
| FAI total score | .99 | .89 to 1.11 | .950 | FAI total score | 1.01 | .90 to 1.12 | .845 | FAI total score | 1.01 | .91 to 1.13 | .839 |
| Ethnicity | .00 | .00 to .00 | .997 | Ethnicity | .00 | .00 to .00 | .997 | Ethnicity | .00 | .00 to .00 | .997 |
| Maternal age birth | 1.01 | .91 to 1.13 | .782 | Maternal age birth | 1.02 | .92 to 1.13 | .705 | Maternal age birth | 1.02 | .92 to 1.13 | .701 |
| Child´s health 4wks | .00 | .00 to .00 | .998 | Child´s health 4wks | .00 | .00 to .00 | .998 | Child´s health4wk | .00 | .00 to .00 | .998 |
| Child´s health 8y | .00 | .00 to .00 | .997 | Child´s health 8y | .00 | .00 to .00 | .997 | Child´s health 8y | .00 | .00 to .00 | .997 |
| Child´s health 10y | .00 | .00 to .00 | .996 | Child´s health 10y | .00 | .00 to .00 | .996 | Child´s health 10y | .00 | .00 to .00 | .996 |
| Child´s health 13y | **8.13** | **1.66 to 40.56** | **.010** | Child´s health 13y | **8.76** | **1.88 to 40.78** | **.006** | Child´s health 13y | **8.74** | **1.88 to 40.72** | **.006** |
|  |  |  |  | **ASTHMA 24 years** | | | |  |  |  |  |
| Anxiety Class1 (ref) | --- | --- | --- | Mood Class2 (ref) | --- | --- | --- | A+D Class2 (ref) | --- | --- | --- |
| Anxiety Class2 | 1.16 | .97 to 1.38 | .114 | Mood Class1 | 1.17 | .91 to 1.50 | .213 | A+D Class1 | 1.14 | .89 to 1.47 | .291 |
| Anxiety Class3 | .98 | .69 to 1.41 | .932 | Mood Class3 | **1.60** | **1.08 to 2.39** | **.020** | A+D Class3 | **1.74** | **1.17 to 2.60** | **.007** |
| Sex | **1.20** | **1.02 to 1.41** | **.024** | Sex | **1.21** | **1.03 to 1.42** | **.018** | Sex | **1.21** | **1.03 to 1.42** | **.018** |
| Gestational age | .96 | .92 to 1.01 | .093 | Gestational age | .97 | .92 to 1.01 | .120 | Gestational age | .97 | .92 to 1.01 | .123 |
| FAI total score | .98 | .96 to 1.00 | .087 | FAI total score | **.98** | **.96 to 1.00** | **.051** | FAI total score | **.98** | **.96 to 1.00** | **.050** |
| Ethnicity | .58 | .27 to 1.27 | .172 | Ethnicity | .58 | .27 to 1.28 | .172 | Ethnicity | .58 | .27 to 1.27 | .175 |
| Maternal age birth | .99 | .98 to 1.01 | .477 | Maternal age birth | .99 | .97 to 1.01 | .334 | Maternal age birth | .99 | .97 to 1.01 | .319 |
| Child´s health 4wks | .00 | .00 to .00 | .998 | Child´s health 4wks | .00 | .00 to .00 | .998 | Child´s health4wk | .00 | .00 to .00 | .998 |
| Child´s health 8y | **2.25** | **1.37 to 3.70** | **.001** | Child´s health 8y | **2.32** | **1.42 to 3.80** | **.001** | Child´s health 8y | **2.32** | **1.42 to 3.80** | **.001** |
| Child´s health 10y | **3.52** | **2.31 to 5.38** | **<.001** | Child´s health 10y | **3.43** | **2.24 to 5.23** | **<.001** | Child´s health 10y | **3.42** | **2.24 to 5.23** | **<.001** |
| Child´s health 13y | .82 | .40 to 1.67 | .583 | Child´s health 13y | .80 | .39 to 1.63 | .534 | Child´s health 13y | .78 | .38 to 1.60 | .504 |

| **ARTHRITIS at 24 years** | | | | | | | | | | | |
| --- | --- | --- | --- | --- | --- | --- | --- | --- | --- | --- | --- |
| **DAWBA ANXIETY** | | | | **DAWBA DEPRESSION** | | | | **DAWBA ANXIETY + DEPRESSION** | | | |
|  | OR | 95% CI | p |  | OR | 95% CI | p |  | OR | 95% CI | p |
| Anxiety Class1 (ref) | --- | --- | --- | Mood Class2 (ref) | --- | --- | --- | A+D Class2 (ref) | --- | --- | --- |
| Anxiety Class2 | **2.75** | **1.46 to 5.15** | **.002** | Mood Class1 | .00 | .00 to .00 | .991 | A+D Class1 | .00 | .00 to .00 | .991 |
| Anxiety Class3 | .00 | .00 to .00 | .993 | Mood Class3 | **6.66** | **2.67 to 16.61** | **<.001** | A+D Class3 | **7.96** | **3.15 to 20.13** | **<.001** |
| Sex | **.42** | **.21 to .81** | **.010** | Sex | **.400** | **.20 to .78** | **.007** | Sex | **.39** | **.20 to .76** | **.006** |
| Gestational age | **.78** | **.68 to .89** | **<.001** | Gestational age | **.78** | **.69 to .89** | **<.001** | Gestational age | **.78** | **.69 to .89** | **<.001** |
| FAI total score | **.85** | **.78 to .94** | **.001** | FAI total score | **.86** | **.78 to .95** | **.003** | FAI total score | **.86** | **.78 to .95** | **.002** |
| Ethnicity | .00 | .00 to .00 | .996 | Ethnicity | .00 | .00 to .00 | .996 | Ethnicity | .00 | .00 to .00 | .996 |
| Maternal age birth | **.81** | **.74 to .89** | **<.001** | Maternal age birth | **.80** | **.73 to .87** | **<.001** | Maternal age birth | **.79** | **.72 to .87** | **<.001** |
| Child´s health 4wks | .00 | .00 to .00 | .998 | Child´s health 4wks | .00 | .00 to .00 | .998 | Child´s health4wk | .00 | .00 to .00 | .998 |
| Child´s health 8y | .00 | .00 to .00 | .997 | Child´s health 8y | .00 | .00 to .00 | .996 | Child´s health 8y | .00 | .00 to .00 | .997 |
| Child´s health 10y | .00 | .00 to .00 | .995 | Child´s health 10y | .00 | .00 to .00 | .994 | Child´s health 10y | .00 | .00 to .00 | .995 |
| Child´s health 13y | **43.6** | **17.5 to 108.3** | **<.001** | Child´s health 13y | **56.2** | **23.0 to 137.2** | **<.001** | Child´s health 13y | **43.6** | **17.5 to 108.3** | **<.001** |
|  |  |  |  | **STROKE / CANCER at 24 years** | | | |  |  |  |  |
| Anxiety Class1 (ref) | --- | --- | --- | Mood Class2 (ref) | --- | --- | --- | A+D Class2 (ref) | --- | --- | --- |
| Anxiety Class2 | 2.21 | .44 to 11.07 | .333 | Mood Class1 | .00 | .00 to .00 | .992 | A+D Class1 | 0.00 | 0.00 to 0.00 | 0.992 |
| Anxiety Class3 | .00 | .00 to .00 | .993 | Mood Class3 | .00 | .00 to .00 | .995 | A+D Class3 | 0.00 | 0.00 to 0.00 | 0.995 |
| Sex | 1.15 | .23 to 5.71 | .863 | Sex | 1.07 | .21 to 5.29 | .937 | Sex | 1.06 | .21 to 5.28 | .939 |
| Gestational age | **2.75** | **1.26 to 5.97** | **.011** | Gestational age | **2.66** | **1.28 to 5.53** | **.009** | Gestational age | **2.66** | **1.28 to 5.52** | **.009** |
| FAI total score | .99 | .80 to 1.22 | .924 | FAI total score | .99 | .80 to 1.24 | .963 | FAI total score | .99 | .80 to 1.24 | .966 |
| Ethnicity | .00 | .00 to .00 | .997 | Ethnicity | .00 | .00 to .00 | .997 | Ethnicity | .00 | .00 to .00 | .997 |
| Maternal age birth | 1.02 | .85 to 1.23 | .822 | Maternal age birth | 1.02 | .85 to 1.23 | .815 | Maternal age birth | 1.02 | .85 to 1.23 | .812 |
| Child´s health 4wks | .00 | .00 to .00 | .998 | Child´s health 4wks | .00 | .00 to .00 | .998 | Child´s health4wk | .00 | .00 to .00 | .998 |
| Child´s health 8y | .00 | .00 to .00 | .997 | Child´s health 8y | .00 | .00 to .00 | .997 | Child´s health 8y | .00 | .00 to .00 | .997 |
| Child´s health 10y | .00 | .00 to .00 | .997 | Child´s health 10y | .00 | .00 to .00 | .996 | Child´s health 10y | .00 | .00 to .00 | .997 |
| Child´s health 13y | .00 | .00 to .00 | .997 | Child´s health 13y | .00 | .00 to .00 | .997 | Child´s health 13y | .00 | .00 to .00 | .997 |

**Table S7b.** Associations between LCGA classes and individual physical health problems at 24 years

**Table S7c.** Associations between LCGA classes and individual physical health problems at 24 years

| **KIDNEY DISEASE at 24 years** | | | | | | | | | | | |
| --- | --- | --- | --- | --- | --- | --- | --- | --- | --- | --- | --- |
| **DAWBA ANXIETY** | | | | **DAWBA DEPRESSION** | | | | **DAWBA ANXIETY + DEPRESSION** | | | |
|  | OR | 95% CI | p |  | OR | 95% CI | p |  | OR | 95% CI | p |
| Anxiety Class1 (ref) | --- | --- | --- | Mood Class2 (ref) | --- | --- | --- | A+D Class2 (ref) | --- | --- | --- |
| Anxiety Class2 | .00 | .00 to .00 | .986 | Mood Class1 | .00 | .00 to .00 | .991 | A+D Class1 | .00 | .00 to .00 | .991 |
| Anxiety Class3 | .00 | .00 to .00 | .994 | Mood Class3 | .00 | .00 to .00 | .995 | A+D Class3 | .00 | .00 to .00 | .995 |
| Sex | 3.65 | .67 to 19.77 | .133 | Sex | 3.09 | .56 to 16.95 | .193 | Sex | 3.09 | .56 to 16.94 | .193 |
| Gestational age | .84 | .61 to 1.15 | .273 | Gestational age | .83 | .60 to 1.15 | .268 | Gestational age | .83 | .60 to 1.15 | .268 |
| FAI total score | **1.21** | **1.10 to 1.34** | **<.001** | FAI total score | **1.20** | **1.10 to 1.31** | **<.001** | FAI total score | **1.20** | **1.10 to 1.31** | **<.001** |
| Ethnicity | .00 | .00 to .00 | .997 | Ethnicity | .00 | .00 to .00 | .997 | Ethnicity | .00 | .00 to .00 | .997 |
| Maternal age birth | 1.02 | .89 to 1.17 | .779 | Maternal age birth | .98 | .85 to 1.12 | .745 | Maternal age birth | .98 | .86 to 1.17 | .748 |
| Child´s health 4wks | .00 | .00 to .00 | .998 | Child´s health 4wks | .00 | .00 to .00 | .998 | Child´s health4wk | .00 | .00 to .00 | .998 |
| Child´s health 8y | .00 | .00 to .00 | .997 | Child´s health 8y | .00 | .00 to .00 | .997 | Child´s health 8y | .00 | .00 to .00 | .997 |
| Child´s health 10y | .00 | .00 to .00 | .996 | Child´s health 10y | .00 | .00 to .00 | .997 | Child´s health 10y | .00 | .00 to .00 | .996 |
| Child´s health 13y | .00 | .00 to .00 | .997 | Child´s health 13y | .00 | .00 to .00 | .997 | Child´s health 13y | .00 | .00 to .00 | .997 |
|  |  |  |  | **HEART PROBLEMS at 24 years** | | | |  |  |  |  |
| Anxiety Class1 (ref) | **---** | **---** | --- | Mood Class2 (ref) | **---** | **---** | --- | A+D Class2 (ref) | **---** | **---** | --- |
| Anxiety Class2 | **.64** | **.44 to .92** | **.017** | Mood Class1 | **1.74** | **1.17 to 2.59** | **.006** | A+D Class1 | .25 | .62 to 1.04 | .056 |
| Anxiety Class3 | **.44** | **.20 to .97** | **.042** | Mood Class3 | **1.24** | **1.06 to 2.98** | **.047** | A+D Class3 | **1.70** | **1.14 to 2.53** | **.008** |
| Sex | 1.07 | .80 to 1.42 | .649 | Sex | 1.08 | .81 to 1.44 | .584 | Sex | 1.08 | .81 to 1.44 | .590 |
| Gestational age | 1.02 | .94 to 1.11 | .620 | Gestational age | 1.02 | .94 to 1.11 | .589 | Gestational age | 1.02 | .94 to 1.11 | .591 |
| FAI total score | .99 | .97 to 1.03 | .966 | FAI total score | .99 | .96 to 1.03 | .693 | FAI total score | .99 | .96 to 1.03 | .703 |
| Ethnicity | .00 | .00 to .00 | .996 | Ethnicity | .00 | .00 to .00 | .996 | Ethnicity | .00 | .00 to .00 | .996 |
| Maternal age birth | .98 | .95 to 1.02 | .343 | Maternal age birth | .98 | .95 to 1.01 | .285 | Maternal age birth | .98 | .95 to 1.02 | .300 |
| Child´s health 4wks | .00 | .00 to .00 | .998 | Child´s health 4wks | .00 | .00 to .00 | .998 | Child´s health4wk | .00 | .00 to .00 | .998 |
| Child´s health 8y | .00 | .00 to .00 | .996 | Child´s health 8y | .00 | .00 to .00 | .996 | Child´s health 8y | .00 | .00 to .00 | .996 |
| Child´s health 10y | .95 | .39 to 2.31 | .913 | Child´s health 10y | .91 | .38 to 2.21 | .843 | Child´s health 10y | .92 | .38 to 2.21 | .849 |
| Child´s health 13y | **10.7** | **6.01 to 19.08** | **<.001** | Child´s health 13y | **1.09** | **6.1 to 19.4** | **<.001** | Child´s health 13y | **10.8** | **6.11 to 19.28** | **<.001** |

**Table S7d.** Associations between LCGA classes and individual physical health problems at 24 years

| **Obesity at 24 years** | | | | | | | | | | | |
| --- | --- | --- | --- | --- | --- | --- | --- | --- | --- | --- | --- |
| **DAWBA ANXIETY** | | | | **DAWBA DEPRESSION** | | | | **DAWBA ANXIETY + DEPRESSION** | | | |
|  | OR | 95% CI | p |  | OR | 95% CI | p |  | OR | 95% CI | p |
| Anxiety Class1 (ref) | --- | --- | **---** | Mood Class2 (ref) | **---** | **---** | --- | A+D Class2 (ref) | --- | --- | **---** |
| Anxiety Class2 | 0.95 | 0.82 to 1.09 | 0.473 | Mood Class1 | 0.95 | 0.77 to 1.18 | 0.643 | A+D Class1 | 1.09 | 0.89 to 1.34 | 0.418 |
| Anxiety Class3 | 1.10 | 0.87 to 1.39 | 0.410 | Mood Class3 | 1.17 | 0.93 to 1.48 | 0.186 | A+D Class3 | **1.30** | **1.01 to 1.66** | **0.039** |
| Sex | **0.40** | **0.36 to 0.45** | **<0.001** | Sex | **0.42** | **0.38 to 0.48** | **<0.001** | Sex | **0.44** | **0.39 to 0.49** | **<0.001** |
| Gestational age | **1.05** | **1.02 to 1.09** | **0.002** | Gestational age | **1.05** | **1.02 to 1.09** | **0.003** | Gestational age | **1.05** | **1.02 to 1.09** | **0.002** |
| FAI total score | **1.01** | **1.00 to 1.02** | **0.038** | FAI total score | **1.01** | **1.00 to 1.02** | **0.035** | FAI total score | **1.01** | **1.00 to 1.02** | **0.020** |
| Ethnicity | 1.04 | 0.68 to 1.59 | 0.864 | Ethnicity | 0.93 | 0.61 to 1.42 | 0.739 | Ethnicity | 0.90 | 0.59 to 1.37 | 0.612 |
| Maternal age birth | 0.99 | 0.98 to 1.00 | 0.076 | Maternal age birth | 0.99 | 0.98 to 1.00 | 0.134 | Maternal age birth | 0.99 | 0.98 to 1.00 | 0.248 |
| Child´s health 4wks | 0.87 | 0.22 to 3.46 | 0.840 | Child´s health 4wks | 0.83 | 0.21 to 3.29 | 0.788 | Child´s health 4wks | 0.83 | 0.21 to 3.29 | 0.787 |
| Child´s health 8y | 0.90 | 0.49 to 1.68 | 0.753 | Child´s health 8y | 0.86 | 0.46 to 1.60 | 0.639 | Child´s health 8y | 0.86 | 0.46 to 1.60 | 0.636 |
| Child´s health 10y | **1.99** | **1.32 to 3.03** | **0.001** | Child´s health 10y | **1.94** | **1.28 to 2.94** | **0.002** | Child´s health 10y | **1.94** | **1.28 to 2.94** | **0.002** |
| Child´s health 13y | 0.59 | 0.31 to 1.10 | 0.098 | Child´s health 13y | 0.61 | 0.33 to 1.13 | 0.118 | Child´s health 13y | 0.60 | 0.32 to 1.13 | 0.113 |

**Table S8.** Associations between LCGA classes and sleep problems at 24 years

* Having problems getting to sleep or back to sleep at 24 years

| **Sleep problems at 24 years*** | | | | | | | | | | | |
| --- | --- | --- | --- | --- | --- | --- | --- | --- | --- | --- | --- |
| **DAWBA ANXIETY** | | | | **DAWBA DEPRESSION** | | | | **DAWBA ANXIETY + DEPRESSION** | | | |
|  | OR | 95% CI | p |  | OR | 95% CI | p |  | OR | 95% CI | p |
| Anxiety Class1 (ref) | --- | --- | --- | Mood Class2 (ref) | **---** | **---** | --- | A+D Class2 (ref) | --- | --- | --- |
| Anxiety Class2 | 1.03 | .94 to 1.14 | .499 | Mood Class1 | **1.20** | **1.05 to 1.38** | **.009** | A+D Class1 | 1.14 | .99 to 1.31 | .062 |
| Anxiety Class3 | .93 | .76 to 1.14 | .494 | Mood Class3 | 1.23 | .98 to 1.54 | .074 | A+D Class3 | **1.35** | **1.07 to 1.71** | **.011** |
| Sex | **.70** | **.64 to .76** | **<.001** | Sex | **.68** | **.62 to .74** | **<.001** | Sex | **.70** | **.64 to .76** | **<.001** |
| Gestational age | **.95** | **.93 to .97** | **<.001** | Gestational age | **.96** | **.94 to .98** | **.001** | Gestational age | **.96** | **.94 to .98** | **<.001** |
| FAI total score | **1.02** | **1.01 to 1.03** | **<.001** | FAI total score | **1.02** | **1.01 to 1.03** | **<.001** | FAI total score | **1.02** | **1.01 to 1.03** | **<.001** |
| Ethnicity | **1.84** | **1.37 to 2.48** | **<.001** | Ethnicity | **1.78** | **1.33 to 2.38** | **<.001** | Ethnicity | **1.64** | **1.22 to 2.20** | **.001** |
| Maternal age birth | .99 | .98 to 1.00 | .172 | Maternal age birth | .99 | .99 to 1.01 | .604 | Maternal age birth | .99 | .98 to 1.00 | .135 |

**Table S9.** Associations between LCGA classes and individual functioning outcomes at 24 years

| **Not in education, employed or in a training scheme at 24 years** | | | | | | | | | | | |
| --- | --- | --- | --- | --- | --- | --- | --- | --- | --- | --- | --- |
| **DAWBA ANXIETY** | | | | **DAWBA DEPRESSION** | | | | **DAWBA ANXIETY + DEPRESSION** | | | |
|  | OR | 95% CI | p |  | OR | 95% CI | p |  | OR | 95% CI | p |
| Anxiety Class1 (ref) | **---** | **---** | --- | Mood Class2 (ref) | **---** | **---** | --- | A+D Class2 (ref) | **---** | **---** | --- |
| Anxiety Class2 | **1.29** | **1.08 to 1.53** | **.004** | Mood Class1 | **1.97** | **1.60 to 2.41** | **<.001** | A+D Class1 | **1.88** | **1.54 to 2.30** | **<.001** |
| Anxiety Class3 | **1.53** | **1.13 to 2.06** | **.006** | Mood Class3 | **1.95** | **1.39 to 2.73** | **<.001** | A+D Class3 | **2.09** | **1.45 to 2.93** | **<.001** |
| Sex | 1.03 | .88 to 1.19 | .738 | Sex | .97 | .83 to 1.12 | .675 | Sex | .94 | .81 to 1.08 | .381 |
| Gestational age | .97 | .93 to 1.01 | .114 | Gestational age | .97 | .93 to 1.01 | .109 | Gestational age | .98 | .94 to 1.02 | .259 |
| FAI total score | **1.03** | **1.01 to 1.04** | **.001** | FAI total score | **1.02** | **1.00 to 1.04** | **.013** | FAI total score | **1.02** | **1.01 to 1.04** | **.002** |
| Ethnicity | .81 | .45 to 1.48 | .497 | Ethnicity | 1.01 | .58 to 1.74 | .984 | Ethnicity | .99 | .57 to 1.70 | .959 |
| Maternal age birth | 1.00 | .98 to 1.02 | .901 | Maternal age birth | 1.00 | .98 to 1.02 | .905 | Maternal age birth | 1.00 | .99 to 1.02 | .839 |
| **Having difficulties in keeping up with coursework/studies at 24 years** | | | | | | | | | | | |
| Anxiety Class1 (ref) | --- | --- | --- | Mood Class2 (ref) | --- | --- | --- | A+D Class2 (ref) | --- | --- | --- |
| Anxiety Class2 | 1.00 | .64 to 1.56 | .999 | Mood Class1 | 1.29 | .73 to 2.27 | .381 | A+D Class1 | 1.32 | .75 to 2.31 | .328 |
| Anxiety Class3 | .55 | .23 to 1.33 | .188 | Mood Class3 | .00 | .00 to .00 | .997 | A+D Class3 | .00 | .00 to .00 | .997 |
| Sex | **.58** | **.39 to .85** | **.006** | Sex | **.64** | **.43 to .94** | **.022** | Sex | **.75** | **.51 to 1.09** | **.130** |
| Gestational age | .93 | .85 to 1.02 | .129 | Gestational age | **.91** | **.83 to .99** | **.038** | Gestational age | .93 | .85 to 1.02 | .110 |
| FAI total score | **1.08** | **1.03 to 1.13** | **.001** | FAI total score | **1.08** | **1.03 to 1.13** | **.001** | FAI total score | **1.08** | **1.03 to 1.13** | **.001** |
| Ethnicity | .00 | .00 to .00 | .997 | Ethnicity | .00 | .00 to .00 | .998 | Ethnicity | .00 | .00 to .00 | .998 |
| Maternal age birth | 1.00 | .96 to 1.04 | .987 | Maternal age birth | 1.01 | .97 to 1.05 | .666 | Maternal age birth | .98 | .94 to 1.03 | .460 |
|  |  |  |  | **Having difficulties in keeping up with work at 24 years** | | | | |  |  |  |
| Anxiety Class1 (ref) | **---** | **---** | --- | Mood Class2 (ref) | --- | --- | --- | A+D Class2 (ref) | --- | --- | --- |
| Anxiety Class2 | **1.47** | **1.06 to 2.02** | **.020** | Mood Class1 | 1.01 | .62 to 1.63 | .975 | A+D Class1 | .94 | .58 to 1.52 | .811 |
| Anxiety Class3 | **2.45** | **1.49 to 4.03** | **<.001** | Mood Class3 | .57 | .20 to 1.61 | .292 | A+D Class3 | .62 | .22 to 1.73 | .359 |
| Sex | **.73** | **.55 to .97** | **.033** | Sex | **.73** | **.55 to .97** | **.030** | Sex | **.75** | **.57 to .99** | **.045** |
| Gestational age | **.91** | **.85 to .99** | **.024** | Gestational age | **.92** | **.86 to .99** | **.040** | Gestational age | .94 | .87 to 1.01 | .104 |
| FAI total score | **1.03** | **1.00 to 1.06** | **.028** | FAI total score | **1.04** | **1.01 to 1.07** | **.015** | FAI total score | **1.04** | **1.00 to 1.07** | **.006** |
| Ethnicity | 1.99 | .91 to 4.39 | .085 | Ethnicity | 1.95 | .89 to 4.26 | .094 | Ethnicity | 1.92 | .88 to 4.18 | .102 |
| Maternal age birth | 1.03 | .99 to 1.06 | .055 | Maternal age birth | **1.04** | **1.01 to 1.07** | **.011** | Maternal age birth | **1.04** | **1.01 to 1.07** | **.008** |

| **ALCOHOL ABUSE at 24 years** | | | | | | | | | | | |
| --- | --- | --- | --- | --- | --- | --- | --- | --- | --- | --- | --- |
| **DAWBA ANXIETY** | | | | **DAWBA DEPRESSION** | | | | **DAWBA ANXIETY + DEPRESSION** | | | |
|  | OR | 95% CI | p |  | OR | 95% CI | p |  | OR | 95% CI | p |
| Anxiety Class1 (ref) | --- | --- | --- | Mood Class2 (ref) | --- | --- | --- | A+D Class2 (ref) | --- | --- | --- |
| Anxiety Class2 | 0.97 | 0.83 to 1.14 | 0.722 | Mood Class1 | **1.50** | **1.23 to 1.84** | **<0.001** | A+D Class1 | 1.32 | 0.92 to 1.89 | 0.125 |
| Anxiety Class3 | 0.78 | 0.56 to 1.07 | 0.124 | Mood Class3 | 1.19 | 0.83 to 1.70 | 0.339 | A+D Class3 | **1.47** | **1.20 to 1.79** | **<0.001** |
| Sex | **2.45** | **2.12 to 2.83** | **<0.001** | Sex | **2.30** | **2.00 to 2.65** | **<0.001** | Sex | **2.24** | **1.95 to 2.57** | **<0.001** |
| Gestational age | 1.03 | 0.99 to 1.07 | 0.088 | Gestational age | 1.04 | 0.99 to 1.08 | 0.054 | Gestational age | 1.04 | 1.00 to 1.08 | 0.046 |
| FAI total score | **1.03** | **1.01 to 1.04** | **<0.001** | FAI total score | **1.01** | **1.00 to 1.03** | **0.043** | FAI total score | **1.01** | **1.00 to 1.03** | **0.040** |
| Ethnicity | 1.28 | 0.82 to 1.99 | 0.280 | Ethnicity | 1.27 | 0.82 to 1.97 | 0.283 | Ethnicity | 1.27 | 0.82 to 1.96 | 0.291 |
| Maternal age birth | 0.99 | 0.98 to 1.01 | 0.418 | Maternal age birth | 1.00 | 0.98 to 1.01 | 0.951 | Maternal age birth | 1.00 | 0.99 to 1.02 | 0.583 |
| **CANNABIS ABUSE at 24 years** | | | | | | | | | | | |
| Anxiety Class1 (ref) | **---** | **---** | --- | Mood Class2 (ref) | **---** | **---** | --- | A+D Class2 (ref) | **---** | **---** | --- |
| Anxiety Class2 | 1.07 | 0.81 to 1.40 | 0.628 | Mood Class1 | **1.64** | **1.15 to 2.34** | **0.006** | A+D Class1 | 1.14 | 0.67 to 1.95 | 0.621 |
| Anxiety Class3 | 0.88 | 0.53 to 1.48 | 0.636 | Mood Class3 | 0.93 | 0.55 to 1.60 | 0.805 | A+D Class3 | **1.76** | **1.23 to 2.51** | **0.002** |
| Sex | **1.56** | **1.20 to 2.03** | **<0.001** | Sex | **1.49** | **1.16 to 1.92** | **0.002** | Sex | **1.41** | **1.10 to 1.81** | **0.006** |
| Gestational age | 0.99 | 0.94 to 1.06 | 0.972 | Gestational age | 1.01 | 0.95 to 1.07 | 0.834 | Gestational age | 1.01 | 0.95 to 1.07 | 0.777 |
| FAI total score | **1.02** | **1.00 to 1.05** | **0.026** | FAI total score | 1.02 | 0.99 to 1.04 | 0.062 | FAI total score | **1.03** | **1.01 to 1.05** | **0.010** |
| Ethnicity | 0.45 | 0.13 to 1.57 | 0.210 | Ethnicity | 0.40 | 0.11 to 1.37 | 0.142 | Ethnicity | 0.38 | 0.11 to 1.34 | 0.131 |
| Maternal age birth | 1.02 | 0.99 to 1.04 | 0.133 | Maternal age birth | 1.01 | 0.99 to 1.03 | 0.380 | Maternal age birth | 1.03 | 1.01 to 1.05 | 0.013 |

**Table S10.** Associations between LCGA classes and individual substance abuse outcomes at 24 years


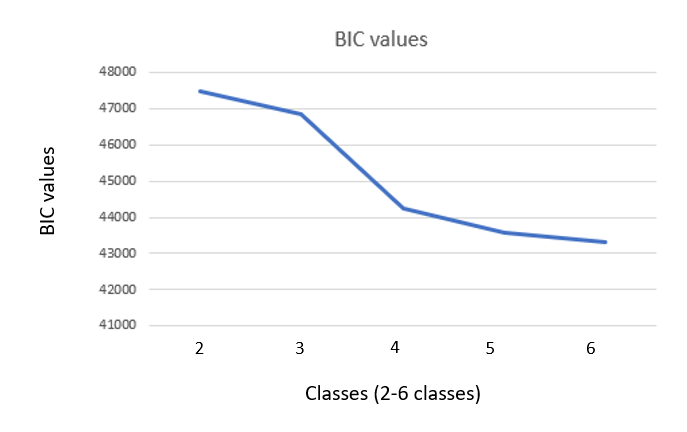


**Fig S1.** BIC values for each of the n-classes model tested using the LCGA (from 2 to 6 classes). The graph shows that BIC peaks within the 3-classes model.
